# Molecular basis of TMED9 oligomerization and entrapment of misfolded protein cargo in the early secretory pathway

**DOI:** 10.1126/sciadv.adp2221

**Published:** 2024-09-20

**Authors:** Le Xiao, Xiong Pi, Alissa C. Goss, Tarick El-Baba, Julian F. Ehrmann, Elizabeth Grinkevich, Silvana Bazua-Valenti, Valeria Padovano, Seth L. Alper, Dominique Carey, Namrata D. Udeshi, Steven A. Carr, Juan Lorenzo Pablo, Carol V. Robinson, Anna Greka, Hao Wu

**Affiliations:** ^1^ Department of Biological Chemistry and Molecular Pharmacology, Harvard Medical School, Boston, MA 02115, USA.; ^2^ Program in Cellular and Molecular Medicine, Boston Children’s Hospital, Boston, MA 02115, USA.; ^3^ Department of Medicine, Brigham and Women’s Hospital and Harvard Medical School, Boston, MA 02115, USA.; ^4^ Broad Institute of MIT and Harvard, Cambridge, MA 02142, USA.; ^5^ Physical and Theoretical Chemistry Laboratory, University of Oxford, Oxford OX1 3QZ, UK.; ^6^ Kavli Institute for Nanoscience Discovery, University of Oxford, Oxford OX1 3QU, UK.; ^7^ Division of Nephrology, Beth Israel Deaconess Medical Center and Department of Medicine, Harvard Medical School, Boston, MA 02215, USA.

## Abstract

Intracellular accumulation of misfolded proteins causes serious human proteinopathies. The transmembrane emp24 domain 9 (TMED9) cargo receptor promotes a general mechanism of cytotoxicity by entrapping misfolded protein cargos in the early secretory pathway. However, the molecular basis for this TMED9-mediated cargo retention remains elusive. Here, we report cryo–electron microscopy structures of TMED9, which reveal its unexpected self-oligomerization into octamers, dodecamers, and, by extension, even higher-order oligomers. The TMED9 oligomerization is driven by an intrinsic symmetry mismatch between the trimeric coiled coil domain and the tetrameric transmembrane domain. Using frameshifted Mucin 1 as an example of aggregated disease-related protein cargo, we implicate a mode of direct interaction with the TMED9 luminal Golgi-dynamics domain. The structures suggest and we confirm that TMED9 oligomerization favors the recruitment of coat protein I (COPI), but not COPII coatomers, facilitating retrograde transport and explaining the observed cargo entrapment. Our work thus reveals a molecular basis for TMED9-mediated misfolded protein retention in the early secretory pathway.

## Teaser

Cryo-EM structures reveal TMED9 oligomerization and interaction with misfolded proteins to exert cytotoxicity and disease.

## INTRODUCTION

Approximately one-third of all newly synthesized polypeptides—especially long and difficult-to-fold membrane proteins—fail to pass the quality control system that ensures proper protein folding. In aging cells, an increased misfolded protein burden contributes to diseases such as Parkinson’s and Alzheimer’s (*1*). Furthermore, hundreds of monogenic proteinopathies arise from mutations in membrane proteins (*2*). For example, Mucin 1 kidney disease (MKD) is caused by a frameshift (fs) mutation in the *MUC1* gene (MUC1-fs) (*3*, *4*). The *MUC1*-encoded transmembrane (TM) glycoprotein is expressed at the apical surface of kidney and other epithelial cells (*5*). Unlike *MUC1*, *MUC1-fs* encodes multiply repeated, Cys-containing unstructured neosequences whose cytotoxic entrapment, primarily in the cis-Golgi and coat protein I (COPI) compartments, ultimately leads to kidney failure. We have reported that TM emp24 domain protein 9 (TMED9) is necessary for MUC1-fs entrapment in MKD, as well as the pathogenesis of additional genetically defined proteinopathies that affect many tissues such as the kidney and the eye (*3*). However, the molecular mechanism by which TMED9 promotes misfolded cargo entrapment remains unknown.

**Fig. 1.**
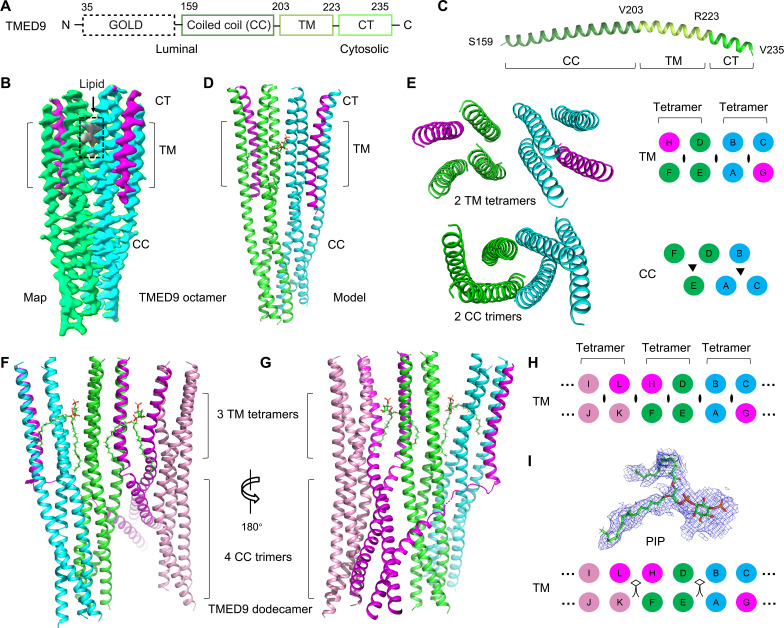
Structures of TMED9 oligomers. (**A**) Domain organization of TMED9. TM, transmembrane helix; CT, cytoplasmic tail. Residue numbers are shown. (**B**) Cryo-EM map of TMED9 octamer with TMED chains in cyan, green, and magenta, and lipid density in gray. For the magenta-colored chains, the coiled coil region is disordered. (**C**) A representative TMED9 subunit structure labeled with the color scheme in (A). (**D**) TMED9 octamer model shown with the color scheme in (B). (**E**) The tetrameric substructures of TMED9 TM domain (top) and the trimeric substructures of TMED9 coiled coli domain (bottom). In the schematic diagrams at right, chain identifications are labeled. The eye-shaped and triangular symbols are C2 and C3 axes, respectively. (**F** to **H**) TMED9 dodecamer model in two orientations [(F) and (G)] and its schematic (H). The four extra TMED9 chains in comparison with the octamer are shown in magenta and pink. Each color in the schematic of the TM arrangement represents a trimer in the coiled coil domain. (**I**) The cryo-EM density and the location of the fitted PIP lipid (indicated by stick figures in the schematic).

TMED9 is a member of the TMED family of cargo receptors that facilitate the bidirectional transport of membrane proteins at the endoplasmic reticulum (ER)–Golgi interface via anterograde COPII and retrograde COPI vesicles (*6*). Sorting signals for COPI (dilysine motif, KK) and COPII (diphenylalanine motif, FF) coatomer proteins (*7*) are contained in the cytosolic tail of TMED9. In addition, all TMEDs share a conserved domain architecture with luminal Golgi-dynamics (GOLD) and coiled-coil (CC) domains and a single TM domain (Fig. 1A) (*6*, *8*). Cargo recognition has been attributed to the GOLD domain (*9*, *10*), but the underlying molecular principles remain unknown. Furthermore, some TMEDs have been shown to either self-dimerize or heterotetramerize (e.g., TMED2/7/9/10) (*11*–*14*). However, the mechanisms of TMED oligomerization remain controversial, and the functional consequences of possible higher-order structures remain unknown.

Here, we report cryo–electron microscopy (cryo-EM) structures of full-length TMED9 in octameric and dodecameric forms. We show that TMED9 self-oligomerization into higher-order structures favors COPI binding relative to COPII binding, to promote retrograde transport and entrapment of misfolded cargo in the early secretory pathway. We reveal that TMED9 recognition of MUC1-fs is mediated by the GOLD domain of TMED9 binding to a predicted MUC1-fs β-strand region located C-terminally to the Cys-containing neosequence repeats of MUC1-fs. These repeats may induce MUC1-fs aggregation in the oxidizing environment of the Golgi. Thus, we uncover a previously unrecognized general mechanism by which higher-order TMED9 oligomers may mediate cargo entrapment in the secretory pathway.

## RESULTS

### Cryo-EM structures of full-length TMED9 reveal the intrinsic symmetry mismatch that promotes higher-order oligomerization

We expressed full-length human TMED9 in Expi293F cells using a FLAG-tagged construct and purified it in *lauryl maltose neopentyl glycol* (*LMNG*) + cholesteryl hemisuccinate (CHS) using anti-FLAG affinity resin and gel filtration chromatography. While previous studies have suggested that TMED9 is a dimer (*13*), the gel filtration elution profile was consistent with a much larger oligomer (fig. S1, A and B), with indication of heterogeneous oligomerization represented by the “ramp” preceding the main TMED9 peak. Solubilization by digitonin or glyco-diosgenin gave a similar size distribution of TMED9 (fig. S1C). By contrast, octyl-β-glucoside, a harsher detergent used previously (*13*, *14*), indeed resulted in elution positions consistent with dimers for both overexpressed TMED9 and TMED2 (fig. S1D). Endogenous TMED9 from kidney epithelial cells (N cells) (*3*, *4*) solubilized in LMNG+CHS migrated similarly as recombinant TMED9 (fig. S1E), ruling out the possibility that the large oligomers are artifacts from overexpression. Thus, by using less harsh detergents, we uncovered TMED9 higher-order oligomerization.

We imaged purified TMED9 using negative staining EM (fig. S1F) and cryo-EM (fig. S1G), and two-dimensional (2D) classification revealed at least two main oligomeric states (fig. S1H). 3D refinement resulted in a structure of TMED9 as an octamer with C2 symmetry at 3.7-Å resolution (Fig. 1, B to E; table S1; and figs. S2 and S3). Further deep 2D and 3D classification revealed another TMED9 structure as a dodecamer at 5.5-Å resolution with no symmetry applied (Fig. 1, F to H; table S1; and figs. S2 and S3). Cryo-EM densities corresponding to the CC domain, the TM domain and the cytosolic tail were clearly visible in the map (Fig. 1B). The GOLD domain exhibited only weak, diffuse density (fig. S4A), likely due to the flexible linker between the GOLD and CC domains. To improve the density for the GOLD domain, we performed various density subtraction and local refinement of the octamer map (fig. S4, B to D). Homogenous refinement of the octamer map after low pass filtering to 30-Å resolution resulted in a map with two additional pieces of density bound to the CC domain that could be fit to the TMED9 GOLD domain model predicted by AlphaFold3 (fig. S4, E and F) (*15*).

An unexpected feature of the TMED9 oligomers is an intrinsic symmetry mismatch between the CC and TM domains. In the homo-octamer, the TM domain consists of a dimer of tetramers, whereas the CC domain forms a dimer of trimers (Fig. 1, D and E); for two of the eight subunits of the TMED9 octamer, the density of the CC domain was absent. In the homo-dodecamer, the TM domain was formed by three tetramers, but the CC domain was formed by four trimers (Fig. 1, F to H). Specifically, we noted that each TM tetramer has internal twofold symmetry, and twofold symmetry is also evident between the TM tetramers (Fig. 1, E and H). In contrast to the symmetry of the TM domain, the CC domain formed obligate trimeric coiled coils, as was also predicted by MultiCoil (*16*). We thus hypothesized that the TM and the cytosolic CC domains are capable of open-ended oligomerization due to the symmetry mismatch, which could explain the ramp in the gel filtration profile (fig. S1, A and B).

We found strong cryo-EM densities between the TM tetramers consistent with phosphatidylinositol phosphates (PIPs) (Fig. 1I and fig. S5). To identify the nature of the PIPs, we performed lipidomics analysis using liquid chromatography−mass spectrometry (LC-MS) (fig. S6 and table S2). The experiment identified a number of PIPs, with PIP (36:2) containing two oleic acyl chains as the most prominent PIP species. PIP (36:2) fits well with the cryo-EM density at both the headgroup and the acyl chains (Fig. 1I). The location of the PIP molecules suggests that they may facilitate TM oligomerization.

### Specific TMED9-TMED9 and TMED9-PIP interactions contribute to self-oligomerization

**Fig. 2.**
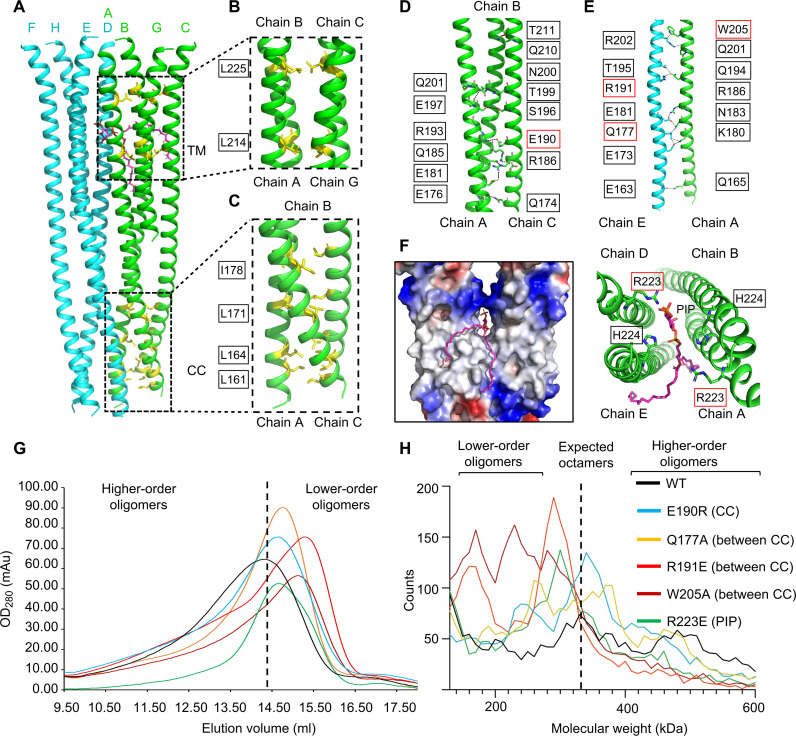
Interactions within TMED9 oligomers. (**A** to **C**) Ile/Leu-mediated hydrophobic interactions (in yellow) within the TM tetramer and coiled coil trimer of TMED9. PIP is in magenta. (**D**) Hydrophilic interactions within the TMED9 coiled coil trimer. (**E**) Hydrophilic interactions between TMED9 coiled coil trimers. (**F**) Hydrophilic interactions between PIP and TMED9 TM domain, as protein surface charge overview (left, in plane of bilayer) and as helical ribbons (right) viewed looking toward the monomeric N termini. (**G**) Gel filtration profile of WT and mutant TMED9. The vertical dashed line marks the peak elution of WT TMED9. (**H**) Mass photometry profile of WT and mutant TMED9. The vertical dashed line marks the expected position of octamers of WT TMED9. Residues mutated here are within red boxes in (D) to (F).

Interactions within the TMED9 oligomers are extensive, totaling ~8000-Å^2^ surface area per subunit as calculated on the PISA server (*17*). Within the TM domain interface, residues L214 and L225 constitute two layers of Leu-mediated hydrophobic interactions (Fig. 2, A and B). Within the CC domain interface, four layers of Ile/Leu-mediated hydrophobic interactions are centered at residues L161, L164, L171, and I178 (Fig. 2, A and C). Hydrogen bonding interactions formed by residues Q174, E176, E181, Q185, R186, E190, R193, S196, E197, T199, N200, Q201, Q210, and T211 likely further stabilize the trimeric CC (Fig. 2D). In addition, several hydrogen bonds may bridge the CC trimers to stabilize the oligomeric assemblies, such as those from E163, Q165, E173, Q177, K180, E181, N183, R186, R191, Q194, T195, Q201, R202, and W205 (Fig. 2E). The PIP headgroup between the TM tetramers is within hydrogen bonding distance to the R223 and H224 side chains from two of the four subunits at the tetramer-tetramer interface (Fig. 2F); these interactions could further stabilize octameric, dodecameric, or higher-order TMED9 complexes. The importance of the observed molecular interactions is supported by delayed elution from the gel filtration column for CC domain mutation E190R, the PIP-interacting residue mutation R223E and CC interface mutations Q177A, R191E, and W205A, all of which compromised TMED9 oligomerization (Fig. 2G). In addition, we used mass photometry, a label-free single-molecule technique for mass determination (*18*), and found that wild-type (WT) TMED9 has a mass distribution consistent with octamers and higher-order oligomers (such as dodecamers) (Fig. 2H). By contrast, the mutants formed smaller oligomers relative to the WT (Fig. 2H), with the magnitude of effect similar to that observed in gel filtration chromatography (Fig. 2G).

### MUC1-fs uses an ordered neo-sequence to bind TMED9 and Cys-containing repeats to aggregate

**Fig. 3.**
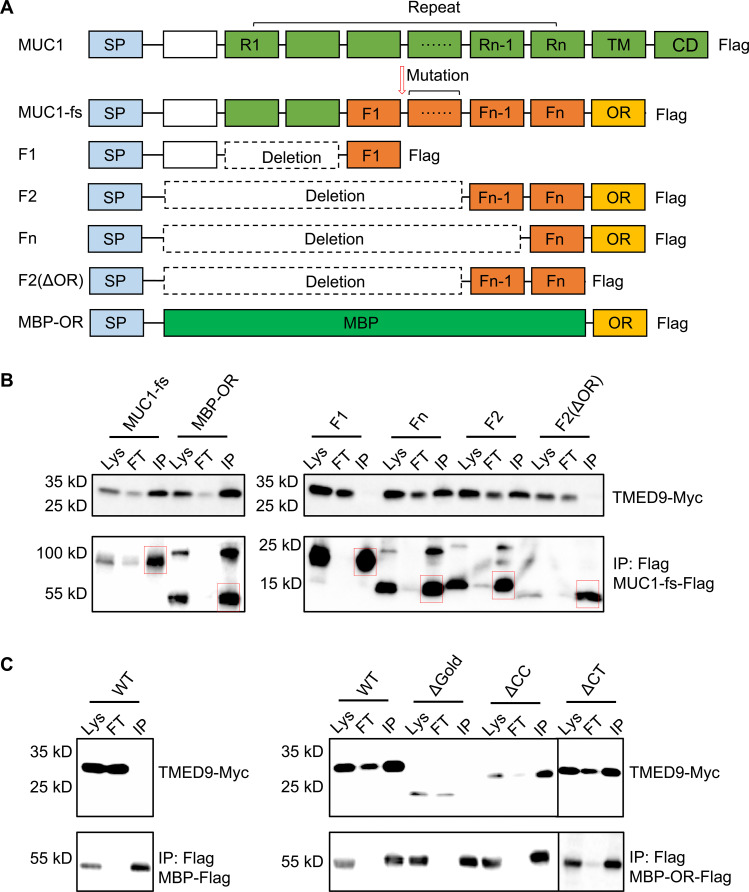
Mapping of the interaction between TMED9 and MUC1-fs. (**A**) Schematic of MUC1 and MUC1-fs constructs. SP, signal peptide; R_1_–R_n_, repeats in MUC1; F_1_–F_n_, repeats in MUC1-fs; OR, ordered region; TM, transmembrane domain; CD, cytoplasmic domain; MBP, maltose-binding protein; Flag, FLAG tag. (**B**) FLAG co-IP of FLAG-tagged MUC1-fs WT and truncation constructs and coexpressed TMED9-Myc. MUC1-fs constructs ran as monomers and/or dimers on reducing SDS-PAGE and immunoblots. Lys, lysate; FT, flow through; IP, IP’ed beads. Bands corresponding to the MW of the MUC1-fs constructs are in red boxes for the IP lane. Some MUC1-fs constructs ran partly as a dimer even in reducing SDS-PAGE, leading to the additional bands. (**C**) FLAG co-IP of FLAG-tagged MBP (negative control, left) or FLAG-tagged MBP-OR (right) with coexpressed WT and single-domain deletions of Myc-tagged TMED9.

Although the TMED GOLD domain is considered responsible for cargo recognition (*9*, *19*), the structural disorder of the GOLD domain in our cryo-EM structures (fig. S4) rendered the structural information unhelpful with respect to the TMED9-cargo interaction. Using the misfolded mutant MUC1-fs neoprotein as a well-established TMED9 cargo (*3*), we sought to understand the molecular underpinnings of its interaction with TMED9. Because WT MUC1 is not entrapped by TMED9 (*3*), we compared the sequences of MUC1-fs and WT MUC1. While WT MUC1 repeats (R_1_-R_n_) lack Cys residues (Fig. 3A and fig. S7A), each neo-repeat (i.e., F_1_-F_n_) of MUC1-fs contains a Cys residue (Fig. 3A and fig. S7B). In addition, while both WT MUC1 and MUC1-fs repeat regions are predicted to contain few secondary structures, the C-terminal region of MUC1-fs just before the neo-stop codon was predicted to consist of two β strands. As this sequence is absent from WT MUC1, we named it the MUC1-fs ordered region (OR) (Fig. 3A).

To elucidate recognition of MUC1-fs by TMED9, we designed MUC1-fs deletion constructs to test by co-immunoprecipitation (IP) the role of repeat and OR sequences in TMED9 interaction. Deletion of repeats did not affect the interaction with TMED9; however, OR deletion dramatically decreased TMED9 interaction (Fig. 3B). All OR-containing constructs avidly co-IP’ed. with TMED9. Moreover, OR alone sufficed for interaction with TMED9 as OR fusion to maltose-binding protein and a FLAG tag (MBP-OR-FLAG) maintained robust interaction with TMED9 (Fig. 3B). Evaluation of TMED9 truncation mutations showed that the GOLD domain is necessary for interaction with the OR of MUC1-fs. Deletion of other TMED9 domains did not alter the interaction between TMED9 and MUC1-fs OR (Fig. 3C).

Since the Cys-containing neo-repeats in MUC1-fs do not contribute to TMED9 interaction, we hypothesized that the role of these repeats might be to promote MUC1-fs aggregation through disulfide bond mispairing within the oxidized environment of the secretory pathway. In support of this notion, MUC1-fs migrated as oligomers on SDS–polyacrylamide gel electrophoresis (SDS-PAGE) in nonreducing conditions but mainly as monomers in reducing conditions (fig. S8). Thus, the MUC1-fs neo-repeats facilitate aggregation, thereby clustering multiple ORs, which may further promote TMED9 oligomerization upon cargo recognition.

### The GOLD domain of TMED9 uses its β-sandwich edge for Muc1-fs interaction

**Fig. 4.**
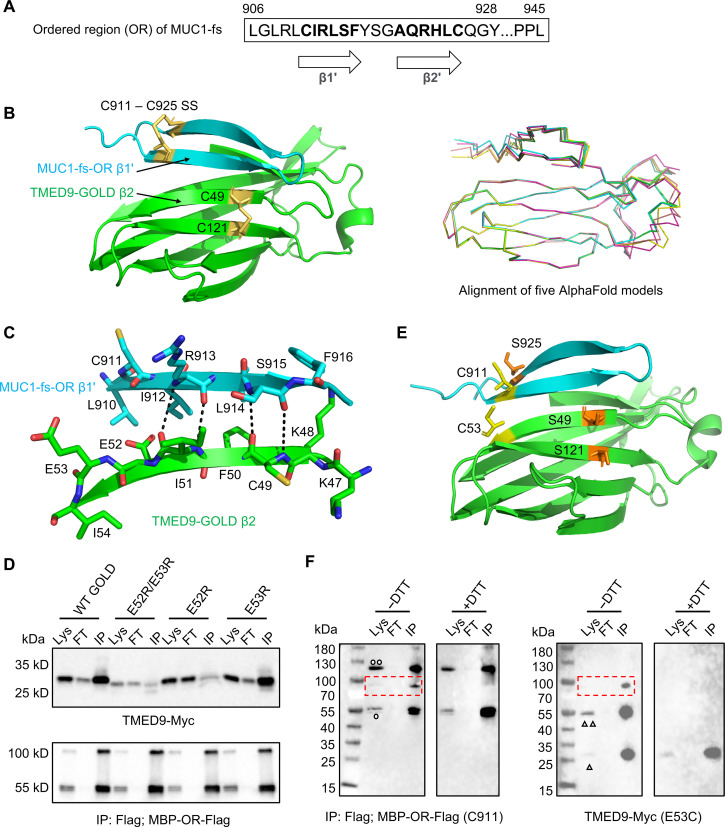
Mode of interaction between MUC1-fs OR and TMED9 GOLD domain. (**A**) Sequence and predicted secondary structure of the OR of MUC1-fs. (**B**) AlphaFold–predicted top-ranked OR-GOLD domain complex model (left) and overlay of the five predicted models (right). In the top-ranked model, TMED9 GOLD domain is in green and MUC1-fs OR is in cyan, and the predicted disulfide bonds are in yellow. (**C**) Interactions are shown between the β1 strand of OR (cyan for carbon atoms) and the β2 strand of GOLD (green for carbon atoms). Other atoms are shown in blue for nitrogen, red for oxygen, and yellow for sulfur. Main chain hydrogen bonds are indicated by black dotted lines. (**D**) FLAG co-IP of FLAG-tagged MBP-OR and Myc-tagged TMED9 WT and mutants. Lys, lysate; FT, flow through; IP, IP’ed. beads. (**E**) OR-GOLD domain complex model with C925 of OR mutated to Ser, C49, and C121 of the GOLD domain mutated to Ser, and E53 of the GOLD domain mutated to Cys, showing the proximity between C911 of OR and C53 of the GOLD domain for possible disulfide bond formation. (**F**) FLAG co-IP of FLAG-tagged C911-only MBP-OR and E53C-only Myc-tagged TMED9. Lys, lysate; FT, flow through; IP, IP’ed. beads. The red dashed boxes indicate disulfide-linked TMED9 and MBP-OR under nonreducing conditions (−DTT) absent under reducing conditions (+DTT). One and two open black circles, respectively, mark the MBP-OR monomer and dimer bands. One and two open black triangles respectively mark TMED9 monomer and dimer bands. MBP-OR migrates as both monomer and dimer even under reducing conditions, for unknown reasons.

Crystal structures of the GOLD domain of TMED1, 2, 5, and 10 (*10*, *20*, *21*) have shown a largely conserved structure, with each GOLD domain consisting of eight β strands to form a β-sandwich fold. One side of the β sandwich, comprising β1 and β2 strands of the GOLD domain, is wide open (fig. S9A), suggesting potential to interact with other β strands by main chain−main chain hydrogen bonding interactions. As the OR of MUC1-fs comprises predicted β strands (β1′ and β2′, Fig. 4A), we wondered whether it could interact with the β1 or β2 strand(s) of the TMED9 GOLD domain. To explore this potential interaction, and because the TMED9 GOLD domain was refractory to crystallization, we used AlphaFold (*22*) to predict the structure of the TMED9 GOLD domain and its complex with the MUC1-fs OR. Predictions generated from each of the five trained models yielded essentially the same structure (Fig. 4B) with a predicted local distance difference test (0 to 100, with 100 being the best) score of 83.9 and a predicted template modeling (0 and 1, with 1 being the best) score of 0.79 for the top-ranked model, suggesting high confidence.

The prediction placed the β1′ strand of the OR in an antiparallel β sheet arrangement with the β2 strand of the TMED9 GOLD domain, with four predicted interstrand main chain hydrogen bonds (Fig. 4C). On the basis of the predicted structure, residues C49 and C121 of the TMED9 GOLD domain form a disulfide bond, in line with the conserved GOLD domain architectures of previously crystalized TMED GOLD domains (*10*, *20*, *21*), and residues C911 and C925 of MUC1-fs also form an intrachain disulfide bond (Fig. 4B). To identify potential key residues in the TMED9 GOLD−MUC1-fs OR interaction, we compared the GOLD domain sequences among different TMEDs and found that residue E52 is conserved (fig. S9B). We thus tested the effect of TMED9 mutations, E52R, E53R, and E52R/E53R on the TMED9 GOLD−MUC1-fs MBP-OR interaction. Both the E52R and E52R/E53R double mutations strongly reduced the interaction of TMED9 with MBP-OR, while E53R had no observable effect on the interaction (Fig. 4D). E52 of TMED9 is in close proximity to R913 of MUC1-fs (Fig. 4C), supporting a charge interaction and explaining the disruptive effects of the E52R mutation.

To further validate the predicted complex structure, we sought to design mutant constructs to test for potential disulfide bond formation between TMED9 and MBP-OR (Fig. 4E). The structures predict proximity between E53 and C911. We mutated TMED9 residues C49 and C121 to Ser and introduced the E53C mutation, thus leaving only one Cys residue in this mutant TMED9. We also mutated MBP-OR residue C925 to Ser, leaving only C911 in this MUC1-fs mutant. When we coexpressed the two proteins and co-IP’ed TMED9 (E53C) using MBP-OR-FLAG (C911), we found that the interaction between the two proteins was preserved (Fig. 4F). Under nonreducing conditions [without dithiothreitol (DTT)], an additional band containing both MUC1-fs and TMED9 was present (Fig. 4F), confirming the proximity of C53 and C911 residues in the mutated TMED9–MUC1-fs protein complex in a manner that enabled disulfide bond formation.

### TMED9 interacts preferentially with COPI but not COPII components

TMED9 uses a conserved KK motif located at the end of its C-terminal cytoplasmic tail to interact with COPI coatomers (*23*). For the COPII coatomer interaction, an FF (diphenylalanine) motif located close to the end of the TM domain has been implicated, as well as the “𝛉C motif” containing a single or a pair of C-terminal hydrophobic residues (*24*–*27*). On the basis of solved structures of COPI coatomers, and specifically of the COPI subunit COPB2 in complex with a TMED9 tail peptide (*23*, *28*), we generated a COPB2-TMED9 complex (fig. S10A) and a modeled TMED9 higher-order oligomer with more than one COPB2 in the context of a COPI coatomer complex (fig. S10B), without steric hindrance. Thus, not only can TMED9 oligomers recruit COPI but they may also exhibit enhanced interaction with COPI through increased avidity. This hypothesis is consistent with previous reports that a higher-order oligomer of the TMED10 cytosolic tail recruits COPI with higher affinity than a dimeric cytosolic tail does (*24*, *29*). By contrast, turning our attention to the TMED9-COPII coatomer interaction, we noted that the conserved FF motif is near the vesicular membrane and buried within the TMED9 oligomer. Only COPII-TMED9 models [based on the structure of COPII subunits SEC24a, SEC23a in complex with the FF motif of ERGIC-53 (*27*)] with TMED9 monomer or dimer were possible, while those with TMED9 tetramer or octamer exhibited steric clash by SEC24a with neighboring subunits of the TMED9 oligomer (fig. S10, C to F), suggesting that TMED9 may not interact with COPII coatomers in its oligomeric form. However, because we do not have structural information to model a 𝛉C motif–based TMED9-COPII coatomer interaction, we cannot exclude the presence of this interaction, nor can we predict the influence of oligomerization on this interaction.

**Fig. 5.**
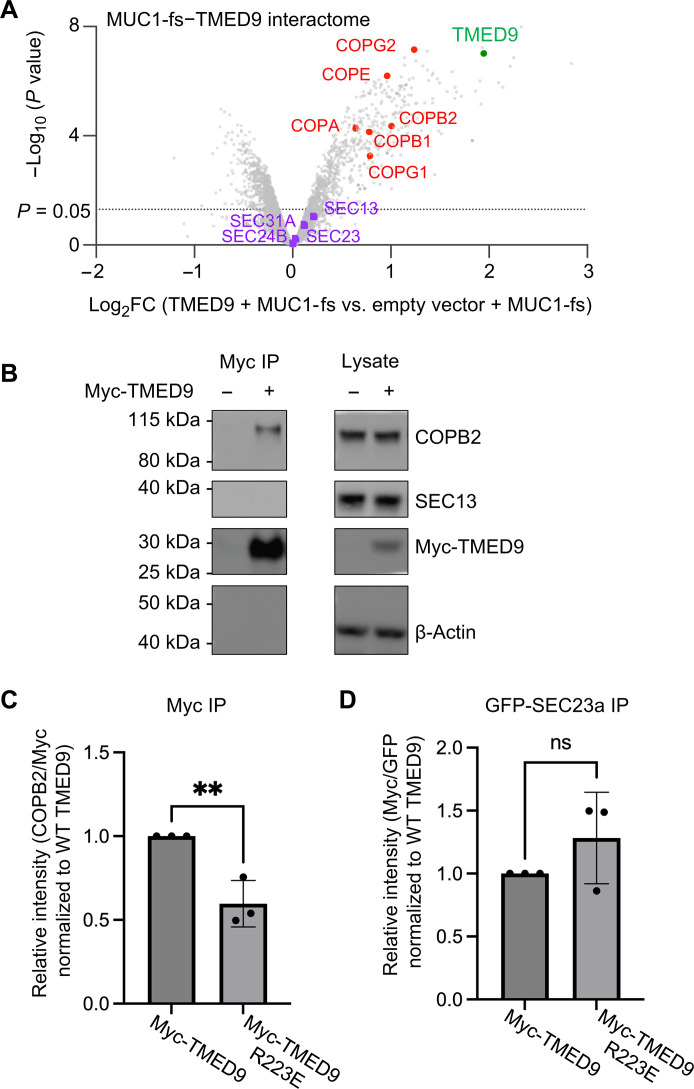
Interaction of TMED9 with COPI but not COPII components. (**A**) Volcano plot of the TMED9 interactome (Myc-TMED9 + MUC1-fs) shows preferential binding of TMED9 (green dot) to COPI proteins (red dots), but not to COPII proteins (purple dots), which did not pass the significance threshold for enrichment compared to the negative control (empty vector + MUC1-fs). (**B**) Myc-tagged TMED9 co-IP’ed. COPB2 (COPI protein) but not SEC13 (COPII protein). β-Actin is shown as a loading control in the lysate and as a negative control in the pulldown. Blots are representative of three independent experiments. (**C**) Myc-tagged TMED9 mutant R223E coimmunoprecipitated less endogenous COPB2 than WT TMED9. (**D**) GFP-tagged SEC23a coimmunoprecipitated mutant and WT TMED9. **P < 0.05. ns., not significant.

To explore putative TMED9 interactors experimentally, we performed IP-MS experiments in human embryonic kidney (HEK) 293 cells co-overexpressing TMED9 and MUC1-fs (receptor + cargo) versus MUC1-fs alone (vector + cargo). These studies revealed preferential association of TMED9 with COPI components but not with COPII components (Fig. 5A). In addition, Myc-tagged TMED9 co-IP’ed endogenous COPB2 of the COPI coatomer but not SEC13 (Fig. 5B). We tested interactions of the COPII component SEC23a or the COPI component COPB2 with WT TMED9 or the TMED9 R223E mutant (which interferes with PIP interaction). To test the interaction with COPB2, we transfected Myc-TMED9 or Myc-TMED9 R223E in HEK293T cells and performed Myc IP. We found a notable decrease of endogenous COPB2 interacting with mutant TMED9 R223E compared with WT TMED9 (Fig. 5C and fig. S11A). This result is consistent with decreased TMED9 oligomerization leading to decreased avidity of interaction with COPB2. In contrast, when we cotransfected green fluorescent protein (GFP)–SEC23a and Myc-TMED9, we observed no significant change in Sec23a binding to WT TMED9 versus the TMED9 R223E mutant (Fig. 5D and fig. S11B). This observation may be explained by our mass photometry and gel filtration data (Fig. 2, G and H), which showed that TMED9 R223E shifts the TMED9 profile to lower-order oligomers but is nevertheless neither a dimer nor a monomer, and, hence, TMED9 R223E does not significantly favor interactions with COPII components (fig. S10, C to F). In sum, these co-IP studies support the notion that higher-order TMED9 oligomerization may be responsible for the avid recruitment of TMED9 to COPI, resulting in entrapment of TMED9-associated aggregated protein cargos such as MUC1-fs in COPI-predominant compartments (COPI vesicles and the cis-Golgi) to prevent their trafficking to and clearance in the lysosome.

## DISCUSSION

**Fig. 6.**
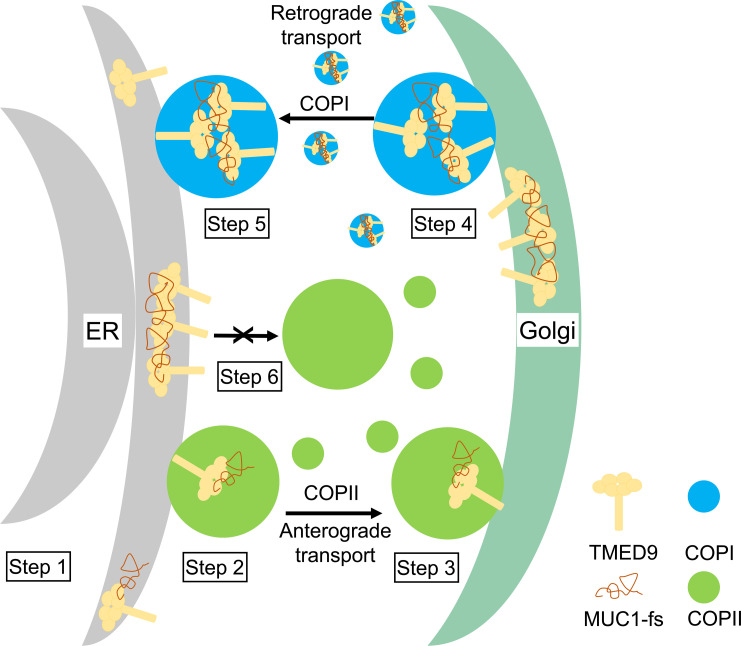
Schematic of the function of TMED9 in mutant cargo accumulation in COPI compartments. Newly expressed MUC1-fs interacts with the GOLD domain of TMED9 (**step 1**) in the ER and is incorporated into the COPII compartment (vesicles or tubules) (**step 2**), which mediate anterograde transport and release into the cis-Golgi (**step 3**). In the cis-Golgi, misfolded MUC1-fs can further oligomerize because of the more oxidizing environment, which may in turn increase interaction of the TMED9–MUC1-fs complex with COPI coatomers (**step 4**). The tripartite COPI-TMED9–MUC1-fs complexes may then attempt to undergo retrograde transport back toward the ER (**step 5**). The higher-order oligomerization state of the TMED9–MUC1-fs complex may inhibit its reentry into the ER, or it may prevent interactions between TMED9 and COPII coatomers (**step 6**). As a result, the misfolded, aggregated MUC1-fs is trapped in COPI-predominant compartments in complex with higher-order oligomeric TMED9.

In this study, our structural and cellular data provide insights into the molecular mechanism by which higher-order oligomers of TMED9 cargo receptors entrap misfolded cargo in the early secretory pathway, leading to cargo accumulation and cellular toxicity. We speculate that the process (Fig. 6) starts with the interaction between MUC1-fs and the GOLD domain of TMED9 within the ER (step 1), leading to their integration into the COPII compartment although inefficiently (vesicles or tubules; step 2). Subsequently, the COPII complex facilitates the forward transport of TMED9–MUC1-fs into the cis-Golgi apparatus (step 3). Within the cis-Golgi, the TMED9–MUC1-fs complex may undergo aggregation in response to the redox environment in the Golgi [more oxidizing than in the ER (*30*)], potentially augmenting interaction with and formation of a tripartite complex with COPI coatomers (step 4). The COPI complex then orchestrates retrograde transport of the substrate, attempting to return it to the ER (step 5). However, the oligomerized TMED9–MUC1-fs complex impedes its efficient entry in the ER (step 6), as ER reentry (and reexit via interaction with COPII coatomers) may necessitate TMED9 to be in a lower-oligomer state. Thus, misfolded MUC1-fs ultimately accumulates in COPI-predominant compartments, where it is entrapped with higher-order TMED9 oligomers. These data reveal the underpinnings of a recently discovered ER-associated degradation escape mechanism for mutant protein handling (*3*) that may be applicable to several genetically defined toxic proteinopathies affecting the kidney, the eye, and other organs.

Our work has led to several important conclusions. First, solving a decades-long puzzle, we determined the full-length structures of a TMED family member. We have detected previously undescribed oligomeric forms of TMED9, including octameric, and dodecameric structures. These and, by extension, additional higher-order self-oligomerizing TMED9 structures, are stabilized by intrinsic symmetry mismatch between the TM and CC domains. The structures suggest residues that are critical for oligomerization, including an unexpected role for the lipid PIP in facilitating this process.

Second, our data suggest that higher-order TMED9 oligomers favor interactions with COPI coatomers, thereby enhancing entrapment of the entire complex in the COPI compartment. While many members of the TMED family have been shown to associate with both COPI and COPII proteomes at steady state (*31*), our data support a specific role for TMED9 oligomers to preferentially bind to COPI coatomers in the presence of misfolded cargo.

Third, our work has broader implications, as TMED9-mediated entrapment of misfolded cargos likely causes many toxic proteinopathies, including uromodulin-associated kidney disease (*32*) and retinitis pigmentosa, a form of blindness (*3*). We identified residues responsible for cargo recognition in the GOLD domain of TMED9, which may provide clues for identifying additional toxic proteinopathies that share the same mechanism of entrapment in the early secretory pathway. For example, mispaired disulfide bond formation contributes to proteinopathies such as cerebral autosomal dominant arteriopathy with subcortical infarcts and leukoencephalopathy (*33*, *34*), neonatal diabetes due to insulin gene mutations (*35*–*37*), and Ehlers-Danlos syndrome due to pro-alpha collagen mutations (*38*). Future studies will likely expand the spectrum of misfolded protein clients. Our work with a highly disordered cargo such as MUC1-fs, and our discovery of an OR that facilitates recognition by TMED9 lead us to further speculate that aggregation of intrinsically disordered regions of misfolded cargos may serve an unanticipated role in facilitating aggregation of ORs to enhance binding and subsequent entrapment by multimeric TMED9 receptors.

## MATERIALS AND METHODS

### Basic TMED9 constructs

Plasmids used were pcDNA3.1(+) plasmid (GenScript), N-terminally tagged MYC TMED9 in pcDNA3.1(+) (referred to as MYC-TMED9), or MYC TMED9 R223E in pcDNA3.1(+) (referred to as MYC-TMED9 R223E). MYC-TMED9 was generated from TMED9 cDNA open reading frame clone (GenScript, OHu00718) via polymerase chain reaction (PCR) using the following primers (fragment 1 generated with TMED9 FW: ATCAATTGCTAGCCCACCATGGCTGTGGAACTGGGAGTG and TMED9 RVMYC: AGACATCTCGAGCTACAGATCCTCTTCA-GAGATGAGTTTCTGCTCCACCAGTTTCTTGGCCTCG; fragment 2 generated with TMED9 FWMYC: ATTACAGCGCCGAGCAGAAA-CTCATCTCTGAAGAGGATCTGCTGTATTTCCACATCG and TMED9 RV: ACGAATCTCGAGTTACACCAGTTTCTTGGCCTCG) using the Q5 Hot Start High-Fidelity DNA Polymerase Kit (New England BioLabs, catalog: M0493L). PCR products were purified via the QIAquick PCR Purification Kit (Qiagen, catalog: 28104) then restriction enzyme digested using CutSmart buffer (New England BioLabs, B7204S, 10091458). Fragment 1 was cut with HaeII (New England BioLabs, catalog: R0107S, lot: 10052648) and XhoI (New England BioLabs, catalog: R0146S, lot: 0581801); fragment 2 was cut with NheI-HF (New England BioLabs, catalog: R3131S, lot: R0146S) and HaeII; and pcDNA3.1(+) was cut with NheI-HF and XhoI. The fragments were ran on an agarose gel (Calbiochem, catalog: C134486, lot: 0655C053) in 1× tris-acetate-EDTA (TAE, Broad Institute SQM, catalog: 50XTAE) at 110 V for 1 hour and 10 min, and then the fragments were excised from the gel and purified via the QIAquick Gel Extraction Kit (Qiagen, catalog: 28706). The purified fragments were then ligated using T4 DNA Ligase (New England BioLabs, catalog: M0202L) at a vector to insert ratio of 1:6 and then transformed into NEB-5α competent cells (New England BioLabs, C2987H) and plated onto 100 μg/ml ampicillin agar plates (Broad Institute SQM, catalog: PET100AMP) and incubated overnight at 37°C. The next day, colonies were picked and grown overnight shaking at 37°C. The following day, the samples were miniprepped and sent for sequencing. The SEC23A plasmid was ordered from Addgene, pEGFP-Sec23A (Addgene, catalog: 66609).

### Transient expression of human TMED9 in Expi293F cells

TMED9 cDNA was cloned into pCDNA3.1 vector with an N-terminal FLAG-tag. Expi293F cells maintained in 800 ml of Expi293 expression medium (Thermo Fisher Scientific) were grown to 2.0 × 10^6^ cells/ml and transiently transfected with 0.8 mg of DNA and 2.4 mg polyethylenimine (PEI, Polysciences Inc.). The cells were fed with 10 mM sodium butyrate and 8 ml of 45% D-(+) glucose solution at 12 hours after transfection. The cells were harvested 48 hours later by 20 min centrifugation at 2500 rpm.

### Purification of full-length TMED9

Sonicated TMED9-expressing cells were resuspended in lysis buffer consisting of 20 mM tris at pH 8.0, 150 mM NaCl, and protease inhibitor cocktails (Roche), and the membrane fraction was collected by centrifugation at 40,000 rpm for 1 hour. The membrane fraction was resuspended in solubilization buffer with 20 mM tris at pH 8.0, 150 mM NaCl, and 1% LMNG-0.1% CHS. After 1 hour incubation at 4°C, solubilized membrane was cleared by centrifugation at 40,000 rpm for 30 min, and the supernatant was incubated with anti-FLAG resin for 1 hour at 4°C with gentle rotation. The resin was then washed with 30 ml of 20 mM tris at pH 8.0, 150 mM NaCl, and 0.02% LMNG-0.002% CHS. The desired protein was eluted with wash buffer containing FLAG (0.1 mg/ml) peptide. The eluted protein was then concentrated for gel filtration using a Superose 6 column. The TMED9-containing peak was collected and concentrated to 1.7 mg/ml [20 mM tris (at pH 8.0), 150 mM NaCl, and 0.02% LMNG+CHS] for cryo-EM grid preparation.

### Cryo-EM grid preparation and data acquisition

Purified TMED9 was loaded onto a glow-discharged Quantifoil grid (R1.2/1.3 400-mesh gold-supported holey carbon, Electron Microscopy Sciences), blotted for 4 s under 100% humidity at 4°C, and plunged into liquid ethane with Mark IV Vitrobot (Thermo Fisher Scientific). For data collection, movies were acquired on a Titan Krios microscope (Thermo Fisher Scientific) at 300 keV equipped with BioQuantum K3 imaging filter (Gatan; slit width, 20 eV). Movies were recorded with a K3 Summit direct electron detector (Gatan) operating in super-resolution mode at 105,000× magnification (0.4125 Å per pixel). All movies were exposed with a flux of 43.2 e^−^/Å^2^/s for 1.36 s fractionated over 43 frames with the defocus range between −1.5 and −2.2 μm.

### Cryo-EM data processing

All data processing computer support was from the SBgrid Consortium (*39*). The first dataset of 4428 movies was corrected by beam-induced motion using the Relion 3.08 (*40*), implementation of the MotionCor2 algorithm (*41*). The contrast transfer function (CTF) and defocus estimation of micrographs without dose-weighting were calculated by CTFFIND4 (*42*). A total of 1,097,397 particles were auto-picked in Relion. These particles were imported into cryoSPARC (*43*) to perform 2D classification, and 530,846 good particles were selected for ab initio reconstruction with K = 5. A total of 707,653 good particles were selected to perform heterogeneous refinement with the above five initial models. A total of 579,405 good particles from three good classes were selected for another heterogenous refinement with four initial models. There were clearly two different structural arrangements in the good classes from the heterogeneous refinement, one with 8 TM helices (octamer) and the other with 12 TM helices (dodecamer). A total of 288,569 particles with the octameric arrangement were selected for nonuniform refinement with C2 symmetry, producing a 4.38-Å resolution map. A total of 171,240 particles with the dodecameric arrangement were selected to carry out nonuniform refinement with C1 symmetry, producing a 6.18-Å resolution map. These good particles in two arrangements were exported into Relion, recentered, and reextracted. CTF refinement and polishing were also performed in Relion, yielding a 4.06-Å resolution map for the octameric arrangement and a 6.03-Å resolution map for the dodecameric arrangement.

The second dataset of 4452 movies was subjected to a similar data processing procedure. A total of 254,434 particles gave a 4.11-Å resolution map for the octamer, and 158,369 particles gave a 5.82-Å resolution map for the dodecamer. Good particles from the two octamer datasets were merged, and 3D classification with local search was performed. Last, 233,493 particles of the octamer were auto-refined in Relion, producing a 3.7-Å resolution map. Good particles from the two dodecamer datasets were merged, and 3D classification with global search was performed. A total of 198,652 dodecamer particles were auto-refined in Relion, yielding a 5.50-Å resolution map. All reported resolutions were estimated on the basis of the gold-standard Fourier shell correlation = 0.143 criterion. All final maps were corrected and sharpened by applying a negative B factor using automated procedures in RELION 3.1. Local resolution variations of cryo-EM maps were estimated using Phenix (*44*).

### Atomic model building and structure representation

The cryo-EM maps were first fit with an AlphaFold–predicted model using UCSF Chimera (*45*), followed by manual adjustment in Coot (*46*), and real-space refinement in Phenix. For all structures, we used PISA5 (*17*) to analyze the interactions. Structure representations were generated in UCSF Chimera and Pymol (*47*).

### Mass photometry

Measurements were performed at the Center for Macromolecular Interactions at Harvard Medical School, on a Refeyn TwoMP atop an Accurion vibration-isolation bench. Proteins were thawed and spun down for 10 min at 21,000 rpm before use. Sample storage buffer contained 0.003% LMNG and 0.0003% CHS. Protein samples were first evenly diluted down to 7 μM concentration in detergent storage buffer. One microliter of the 7 μM sample was diluted again with 4 μl buffer A [25 mM Hepes (pH 7.4), 150 mM KCl, and 1 mM DTT], and 1 μl of this second sample dilution was added directly into the measurement drop comprising 15 μl of buffer A. The drop was pipetted up and down carefully several times, and less than 10 s passed between dilution and measurement. Events were exported and plotted in GraphPad Prism 10 (Dotmatics).

### Lipidomics by MS

TMED9 (50 μg) was digested with trypsin overnight at 37°C to release bound lipids. The liquid was evaporated under reduced pressure using a Speedivac overnight, and the resulting pellet was resuspended in 50:50 methanol:water supplemented with 0.12% (v/v) of 70% ethylamine (lipidomics buffer A) in an ultrasonic bath for ~30 min. Insoluble materials were pelleted at 20,000*g* for 10 min at room temperature. The supernatant was transferred to a round-bottomed glass vial for immediate analysis.

Lipidomics analysis was carried out using a procedure similar to that described by Ogiso and Taguchi (*48*) with some slight modifications. Lipids were separated on an Ultimate 3000 nanoLC system (Thermo Fisher Scientific, San Jose, CA) using a PepMap 100 C8 analytical column (Life Technologies, 3 μm, 0.075 mm by 150 mm) with a binary buffer system consisting of lipidomics buffer A and lipidomics buffer B 100:0.12 isopropyl alcohol:70% ethylamine. One microliter of lipids was loaded onto the column using 95:5 lipidomics buffer A:buffer B at a flow rate of 300 nl min^−1^ for 7.5 min. The lipids were separated by increasing the lipidomics buffer B concentration from 5 to 90% over 10 min, and then the column was washed 5 min with 99% lipidomics buffer B to clear any retained compounds.

The separated lipids were electrosprayed in the negative ion mode (2 to 2.1 kV) into an Orbitrap Eclipse Tribrid mass spectrometer (small-molecule mode, capillary temperature 320°C). Lipid ions were analyzed at a resolving power of 120,000 [at mass/charge ratio (*m*/*z*) 200] for a maximum of 50-ms inject time. Singly and doubly charged precursor ions with a single scan abundance >1.0 × 10^5^ were isolated using the quadrupole (1.5-Th isolation width) and subjected to a stepped higher-energy collisional dissociation (HCD) energy of 25 and 30% to generate fragment ions. The product ions were subsequently analyzed using the Orbitrap mass analyzer operated at a resolving power of 15,000 at *m*/*z* 200 for 50 ms (first mass *m*/*z* 75). Precursors within 10 parts per million (ppm) were dynamically excluded for 30 s. The presence of PIP was determined by manual inspection of MS^2^ spectra for the characteristic signals of the headgroups of inositol phosphate and PIP1 (241.01 and 320.9 Th, respectively).

### TMED9 and MUC1-fs OR co-IP studies

DNA constructs of Myc-TMED9 and FLAG-MUC1-fs were transfected into HEK293T cells with PEI. Transfected cells were lysed with lysis buffer [20 mM tris (at pH 8.0), 150 mM NaCl, and 1% LMNG + 0.1% CHS]. The post-centrifugation supernatant was incubated with anti-FLAG resin for 1 hour at 4°C. The beads were washed extensively and then eluted with FLAG peptide. Eluates were subjected to SDS-PAGE, and gels were transferred to polyvinylidene difluoride membrane with the Trans-Blot Turbo Transfer System (Bio-Rad). Membranes were blocked with 5% nonfat milk for 1 hour at room temperature and then incubated with horseradish peroxidase (HRP)–conjugated primary antibody against FLAG and Myc tags, as indicated.

### TMED9 mutant and COP component co-IP studies

For the MYC Co-IP, 5 × 10^6^ HEK293T cells were plated into a 10-cm plate per condition in Dulbecco’s modified Eagle’s medium (DMEM) (1×) + GlutaMAX (Gibco, catalog: 10569-010) + 10% fetal bovine serum (FBS, Gibco, catalog: 26140-079) + 1% penicillin-streptomycin (Gibco, catalog: 1510-122). The next day, the cells were transfected by combining 600 μl of Opti-MEM (1×) (Gibco, catalog: 31985-070) with 4.34 μg of plasmid DNA, and then in a separate tube, 600 μl of Opti-MEM and 8.68 μl of Lipofectamine 2000 (Invitrogen, catalog: 11668-019) were combined. The Opti-MEM and Lipofectamine was then pipetted and mixed into the Opti-MEM and plasmid DNA tube and incubated at room temperature for 5 min. Fresh medium was added to the 10-cm dishes, and then the full volume of transfection reagent was pipette on the side of the plates and incubated overnight at 37°C with 5% CO_2_. The next day, the plates were placed on ice, washed once with ice-cold phosphate-buffered saline (PBS, Gibco, catalog: 10010-023), and then the cells were scraped into 1 ml of cold co-IP lysis buffer. Co-IP lysis buffer contains 100 mM NaCl (Invitrogen, catalog: AM9759), 5 mM EDTA (Broad Institute SQM, catalog: 05MEDTA), 50 mM tris (pH 7.5) (Invitrogen, catalog: 15567-027), and 1% NP-40 (Thermo Fisher Scientific, catalog: 28324) adjusted to pH 7.5 while cold, and before use, 1 tablet each of protease inhibitors (Roche, catalog: 04693159001) and phosphatase inhibitors (Roche, catalog: 04906837001) are dissolved into the buffer per every 10 ml of the buffer. The scraped cells were rotated for 30 min at 4°C to lyse and then spun at 13000*g* for 15 min at 4°C; then, the supernatant was moved to a new tube and kept on ice. Protein was quantified for each sample using bovine serum albumin standards (Thermo Fisher Scientific, catalog: 23208) and a bicinchoninic acid protein assay reagent A (Thermo Fisher Scientific, catalog: 23228) and reagent B (Thermo Fisher Scientific, catalog: 23224). Using a magnetic rack, 50 μl of well resuspended anti–c-MYC magnetic beads (Thermo Fisher Scientific, catalog: 20169) per sample plus excess were washed thrice with 1 ml of 1× TBST composed of 150 mM NaCl, 50 mM tris (pH 7.5), and 0.05% Tween-20 (Sigma-Aldrich, catalog: P1379-500ML), and then the beads were resuspended in the original volume of co-IP lysis buffer and 50 μl of resuspended beads per sample were aliquoted into new tubes for the co-IP. Next, 1.5 mg of lysate protein was added to the tubes containing beads, and the volume was adjusted to 1 ml using co-IP lysis buffer. The lysate plus beads were rotated for 30 min at room temperature. After 30 min, the tubes were placed onto the magnetic rack and the supernatant was removed and saved as the unbound fraction for quality control. The samples were then washed by adding 1 ml of 5× TBST (750 mM NaCl, 250 mM tris (pH 7.5), and 0.25% Tween-20) to each tube, and then the tubes were removed from the magnetic rack and placed in a standard benchtop tube rack and sandwiched between another tube rack. These sandwiched tubes and tube racks were then shaken 20 times to wash all the samples at once before the samples were placed on the magnetic rack for a minute to allow the beads to be collected, and the wash buffer was removed. The samples were washed three times. After the last wash, the tubes were spun briefly in a microcentrifuge to collect any beads or buffer on the lid, and the tubes were placed on the magnetic rack where all wash buffer was removed via pipetting, and then any residual buffer was removed via aspiration, taking care not to aspirate any beads. The beads are then resuspended in 30 μl of elution buffer containing 2× lithium dodecyl sulfate (LDS) (Life Technologies, catalog: NP0008), 1× DTT (Invitrogen, catalog: NP0009), and water (Life Technologies, catalog: AM90937) and then heated at 75°C for 10 min; these are the co-IP samples. The lysate samples were then prepped for a final concentration of 1 μg/μl in 1× LDS, 1× DTT, and water and heated at 75°C for 10 min. Loaded 25 μl of co-IP samples and lysate samples were loaded onto a 4 to 12% bis-tris gel (Invitrogen, catalog: NP0336BOX) along with PageRuler Preset Ladder (Thermo Fisher Scientific, catalog: 26616). The gel ran in 1× MES SDS buffer (Invitrogen, catalog: NP0002) for 2 hours at 125 V and then was transferred to a blot (Bio-Rad, catalog: 1704158) and stained with Ponceau (Sigma-Aldrich, catalog: P7170-1 L) to check for proper transfer. The blot was cut into several pieces then was blocked for 1 hour in 10% milk (Cell Signaling Technology, catalog: 9999S) in PBST (1% Tween in PBS). The blots were incubated overnight shaking at 4°C with the following antibodies in 5% milk in PBST: 1:1000 dilution of c-MYC (9E10) HRP mouse monoclonal immunoglobulin G (Santa Cruz Biotechnology, catalog: sc-40, lot: L1520), 1:4000 dilution of rabbit anti-COPB2 (Bethyl, catalog: A304-523A), and 1:1000 dilution of β-actin (13E5) rabbit monoclonal antibody HRP conjugate (Cell Signaling Technology, catalog: 5125S). The next day, the blots were washed three times for 10 min with PBST, then Veriblot (Abcam, AB131366) for IP detection reagent was added to the COPB2 blot for 1 hour shaking at room temperature, and then the blots were washed three times for 10 min with PBST. The blots were incubated, shaking at room temperature, with Pico PLUS enhanced chemiluminescence (ECL) reagent (Thermo Fisher Scientific, catalog: 34578) for 5 min, or Femto ECL (Thermo Fisher Scientific, catalog: 34096) for 3 min if needed, and then colorimetric and chemiluminescent images were taken on the basis of the optimal autoexposure on a Bio-Rad ChemiDoc Imaging System.

The GFP co-IP was performed in the same manner as the MYC co-IP except for the following details. During the transfection, we combined 600 μl of Opti-MEM (1×) with 6.51 μg of plasmid DNA in a 1:1 ratio; then, in a separate tube, 600 μl of Opti-MEM and 13.02 μl of Lipofectamine 2000 were combined. GFP-Trap magnetic agarose beads (Chromotek, catalog: gtma-20) were used, and the beads were washed twice with 1× TBST before the addition of lysate and incubated for 1 hour at 4°C before washing two times with 5× TBST. The blots were ran and were incubated except that GFP-HRP (Cell Signaling Technology, catalog: 2037S) was used instead of COPB2.

### P1A8 cell culture

P1A8 (female) immortalized kidney epithelial cells (*3*) were maintained at 37°C with 5% CO_2_ in RenaLife epithelial medium supplemented with RenaLife LifeFactors (Lifeline Cell Technology) without gentamycin and amphotericin B supplement. The cells were passaged at 100% confluency, approximately twice per week, and split at a ratio of 1:3. The medium was changed once between passages. At time of passage (say in a T75 flask), the medium was aspirated and the cells were washed once with 10 ml of PBS, then treated with 5 ml of TrypLE Express Enzyme (1×) without phenol red (Gibco), and incubated at 37°C for 5 to 10 min until cell detachment was evident. The cells were then resuspended in 7 ml of medium and spun down at 400*g* for 5 min. The supernatant was aspirated from the pelleted cells, and the cells were resuspended in medium and plated as desired.

### IP samples for proteomics

For transfection, 8.1 × 10^6^ HEK293T cells were plated onto one 10-cm plate per condition in DMEM (1×) + GlutaMAX (Gibco, catalog: 10569-010) + 10% FBS (Gibco, catalog: 26140-079) + 1% penicillin-streptomycin (Gibco, catalog: 1510-122). The next day, the cells were transfected by first combining 500 μl of Opti-MEM (1×) (Gibco, catalog: 31985-070) with 9.72 μg of total plasmid DNA per condition (MYC-TMED9 and FLAG-MUC1-fs or empty vector and FLAG-MUC1-fs). Then, in separate tubes, 500 μl of Opti-MEM and 19.44 μl of Lipofectamine 2000 (Invitrogen, catalog: 11668-019, lot: 2423710) per condition were combined. The Opti-MEM and DNA mixtures were then pipetted and mixed into the Opti-MEM and Lipofectamine 2000 mixtures and incubated at room temperature for 5 min. Fresh medium was added to the 10-cm dishes, and then the full volume of transfection mixture for each condition was pipetted dropwise into the plates, which were incubated overnight at 37°C in 5% CO_2_. Three biological replicates of each of the two transfection conditions were performed. MYC-TMED9 was immunoprecipitated from each replicate and sent for MS analysis.

For the MYC-TMED9 IPs, cell lysates were prepared as above. Well-resuspended Myc-Trap magnetic agarose beads (37.5 μl; Chromotek, catalog: ytma-20) per condition was washed together three times with 1 ml of lysis buffer. Then, the beads were resuspended to the original volume in lysis buffer and divided among the samples. Three milligrams of lysate protein (2 mg of input protein/25 μl of beads) per condition was added to the tubes containing beads and the volume of each tube adjusted to 2 ml with lysis buffer. The lysate and bead mixtures were rotated for 1 hour at 4°C. The supernatant from each sample was saved and prepared for quality control Western blot analysis. The beads were washed as above, three times with lysis buffer and three times with Chromotek-suggested wash buffer [10 mM tris-HCl (pH 7.5), 150 mM NaCl, and 0.5 mM EDTA (pH 7.5) at 4°C, with one tablet each of protease inhibitors (Roche, catalog: 04693159001) and phosphatase inhibitors (Roche, catalog: 0490683700) dissolved per 10 ml of buffer]. After completing the washes, each bead sample was resuspended in 900 μl of wash buffer and 600 μl (equivalent to 2-mg input protein) was subjected to MS analysis (Broad Institute Proteomics Platform). Protein from the remaining 300 μl of resuspended beads (equivalent to 1 mg of input protein) was eluted by bead suspension in 25 μl of elution buffer containing 2× LDS (Invitrogen, catalog: NP0008), 1× DTT (Invitrogen, catalog: NP0004), and nuclease-free water (Invitrogen, catalog: AM9937), followed by heating at 95°C for 10 min. The eluted protein was saved for quality control Western blot analysis.

### Proteomics sample processing: On-bead trypsin digestion of biotinylated proteins

Proteins bound to antibody beads were washed 4× with 200 μl of 50 mM tris-HCl (pH 7.5). The final wash was removed, and beads were incubated with 80 μl of 2 M urea in 50 mM tris-HCL containing 1 mM DTT and 0.4 μg of trypsin at 25°C for 1 hour while shaking at 1000 r.p.m. After 1 hour, the supernatant was removed and transferred to fresh tubes. The beads were washed twice with 60 μl of 2 M urea in 50 mM tris (pH 7.5) buffer and combined with the on-bead digest supernatant. Disulfide bonds were reduced with 4 mM DTT at 25°C for 30 min with shaking at 1000 r.p.m. Sample eluent was alkylated with 10 mM iodoacetamide at 25°C for 45 min in the dark while shaking at 1000 r.p.m. The samples were digested with 0.5 μg of trypsin overnight at 25°C with shaking at 700 r.p.m. Following overnight digestion, formic acid (FA) was added to eluents to ∼1% (v/v) and pH 3.

Peptides were desalted using C18 StageTips. Briefly, C18 StageTips were conditioned with 100 μl of 100% MeOH, 100 μl of 0.1% (v/v) FA, and 50% (v/v) acetonitrile, and twice with 100 μl of 0.1% (v/v) FA. Acidified peptides were loaded onto the conditioned StageTips and washed twice with 100 μl of 0.1% (v/v) FA. Peptides were eluted from the StageTips with 50 μl 0.1% (v/v) FA and 50% (v/v) acetonitrile and vacuum centrifuged until completely dry.

### Proteomics sample processing: TMT labeling and bRP StageTip fractionation of peptides

For tandem mass tag (TMT) labeling, peptides were reconstituted in 80 μl of 50 mM Hepes. Each sample was labeled with 20 μl of a specific TMT10 (20 mg/ml) label for 1 hour while shaking at 1000 r.p.m. TMT-labeling reactions were quenched with 4 μl of 5% (v/v) hydroxylamine at room temperature for 15 min with shaking and then combined. The samples were dried, and peptides were desalted on C18 StageTips as above.

For each sample, 50% was fractionated by basic pH reversed-phase (bRP) fractionation. A StageTip was prepared using two disks of SDB-XC material (Empore 2240), washed, and equilibrated with 100% MeOH, followed by 50% acetonitrile (ACN)/1% FA and 0.1% FA. Dried peptides were resuspended in 3% FA/5% ACN and loaded onto the StageTip. Peptides were eluted in six fractions with increasing concentrations of ACN (5, 10, 15, 20, 25, and 45%) in 0.1% (w/v) NH_4_OH [28% NH_3_ w/v)], pH 10. Fractions were dried down in a vacuum concentrator.

### MS data processing

Desalted, TMT-labeled peptides were resuspended in 9 μl of 3% MeCN and 0.1% FA and analyzed by online nanoflow LC tandem MS (LC-MS/MS) using an Exploris 480 (Thermo Fisher Scientific) coupled on-line to a Proxeon Easy-nLC 1200 (Thermo Fisher Scientific). Four microliters of each sample was loaded at 500 nl/min onto a microcapillary column (360 μm outer diameter × 75 μm inner diameter) containing an integrated electrospray emitter tip (10 μm), packed to approximately 24 cm with ReproSil-Pur C18-AQ 1.9 μm beads (Dr. Maisch GmbH) and heated to 50°C. The high-performance LC solvent A was 3% MeCN and 0.1% FA, and the solvent B was 90% MeCN and 0.1% FA. The peptides were eluted into the mass spectrometer at a flow rate of 200 nl/min. Nonfractionated samples were analyzed using a 154-min LC-MS/MS method with the following gradient profile: (min:%B) 0:2; 2:6; 120:35; 122:60; 130:90; 143:90; 144:50; and 154:50 (the last two steps at 500 nl/min flow rate). The bRP fractions were run with the 110-min method using the following gradient profile: (min:%B) 0:2; 1:6; 85:30; 94:60; 95:90;100:90; 101:50; and 110:50 (the last two steps at 500 nl/min flow rate). The Exploris 480 was operated in the data-dependent mode acquiring HCD MS/MS scans (*r* = 45,000) after each MS1 scan (*r* = 60,000) on the top 20 most abundant ions using an MS1 target of 3E6 and an MS2 target of 5E4. The maximum ion time used for MS/MS scans was 120 ms (single-shot) and 105 ms (bRP fractions); the HCD normalized collision energy was set to 32; the isolation window (*m*/*z*) = 0.7; the dynamic exclusion time was set to 20 s, and the peptide match and isotope exclusion functions were enabled. Fit filter was enabled at 50% using a purity window of 1.2.

### MS data analysis

Collected data were analyzed using the Spectrum Mill software package v6.1 prerelease (Agilent Technologies). Nearby MS scans with similar precursor *m*/*z* were merged if within ±45-s retention time and ±1.4-*m*/*z* tolerance. MS/MS spectra were excluded from searching if they failed the quality filter by lacking sequence tag length 0 or precursor MH+ in the range of 600 to 6000. All extracted spectra were searched against a UniProt human database. Search parameters included ESI QEXACTIVE-HCD-v2 scoring parent and fragment mass tolerance of 20 ppm, 30% minimum matched peak intensity, trypsin allow P enzyme specificity with up to four missed cleavages, and calculated reversed database scores enabled. Fixed modifications were carbamidomethylation at cysteine. TMT labeling was required at lysine, but peptide N termini were allowed to be either labeled or unlabeled. Allowed variable modifications were protein N-terminal acetylation and oxidized methionine. Individual spectra were automatically assigned a confidence score using the Spectrum Mill autovalidation module. Score at the peptide mode was based on target-decoy false discovery rate of 1%. A second round of validation was performed at the protein level, requiring a minimum protein score of 0. Relative abundances of proteins were determined using TMT reporter ion intensity ratios from each MS/MS spectrum, and the median ratio was calculated from all MS/MS spectra contributing to a protein subgroup. Proteins identified by two or more distinct peptides and ratio counts were considered for the dataset.
