# Supplementary material for: Molecular basis of TMED9 oligomerization and entrapment of misfolded protein cargo in the early secretory pathway
Source: Sci Adv. 2024 Sep 20;10(38):eadp2221. doi: 10.1126/sciadv.adp2221 (PMC11414720; doi:10.1126/sciadv.adp2221)
Supplement: Supplementary file 1 — Figs. S1 to S11 Tables S1 and S2 [file sciadv.adp2221_sm.pdf]

Science Advances
AAAS

Supplementary Materials for

Molecular basis of TMED9 oligomerization and entrapment of misfolded protein cargo in the early secretory pathway

Le Xiao *et al.*

Corresponding author: Hao Wu, wu@crystal.harvard.edu; Anna Greka, agreka@bwh.harvard.edu

Sci. Adv. **10**, eadp2221 (2024)
DOI: 10.1126/sciadv.adp2221

This PDF file includes:

Figs. S1 to S11
Tables S1 and S2

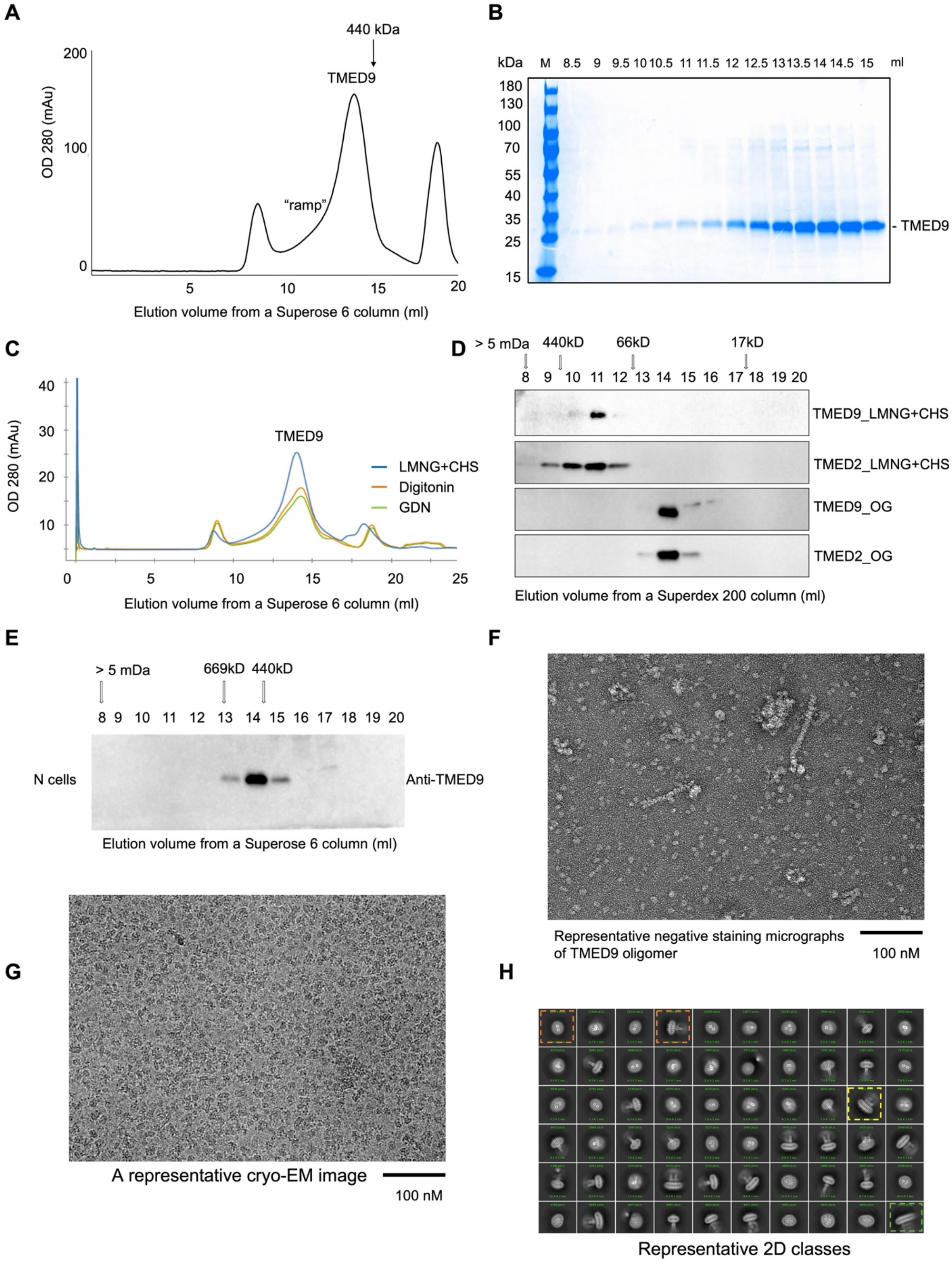

Fig. S1. Cryo-EM sample preparation of TMED9. (A-B) Gel filtration profile using a Superose 6 column (A**)** and SDS-PAGE for the main peak fraction (B) of TMED9 during the purification. **(C)**

TMED9 purified in different detergents showed similar gel filtration profiles. **(D)** WB for TMED9 and TMED2 in different detergents after gel filtration with Superdex 200, showing that OG, a harsher detergent used previously(*13, 14*), resulted in elution positions consistent with TMED9 or TMED2 dimers**. (E)** WB for endogenous TMED9 from N cells after gel filtration with Superose 6 using LMNG+CHS as the detergent + lipid mix. **(F)** A representative negative staining micrograph of TMED9 oligomer from (A). **(G)** A representative cryo-EM micrograph from (A). **(H)** Representative 2D class averages. The orange squares indicate 2D classes of octameric TMED9. The yellow square indicates a 2D class of dodecameric TMED9. The green square indicates a 2D class of higher oligomer of TMED9.

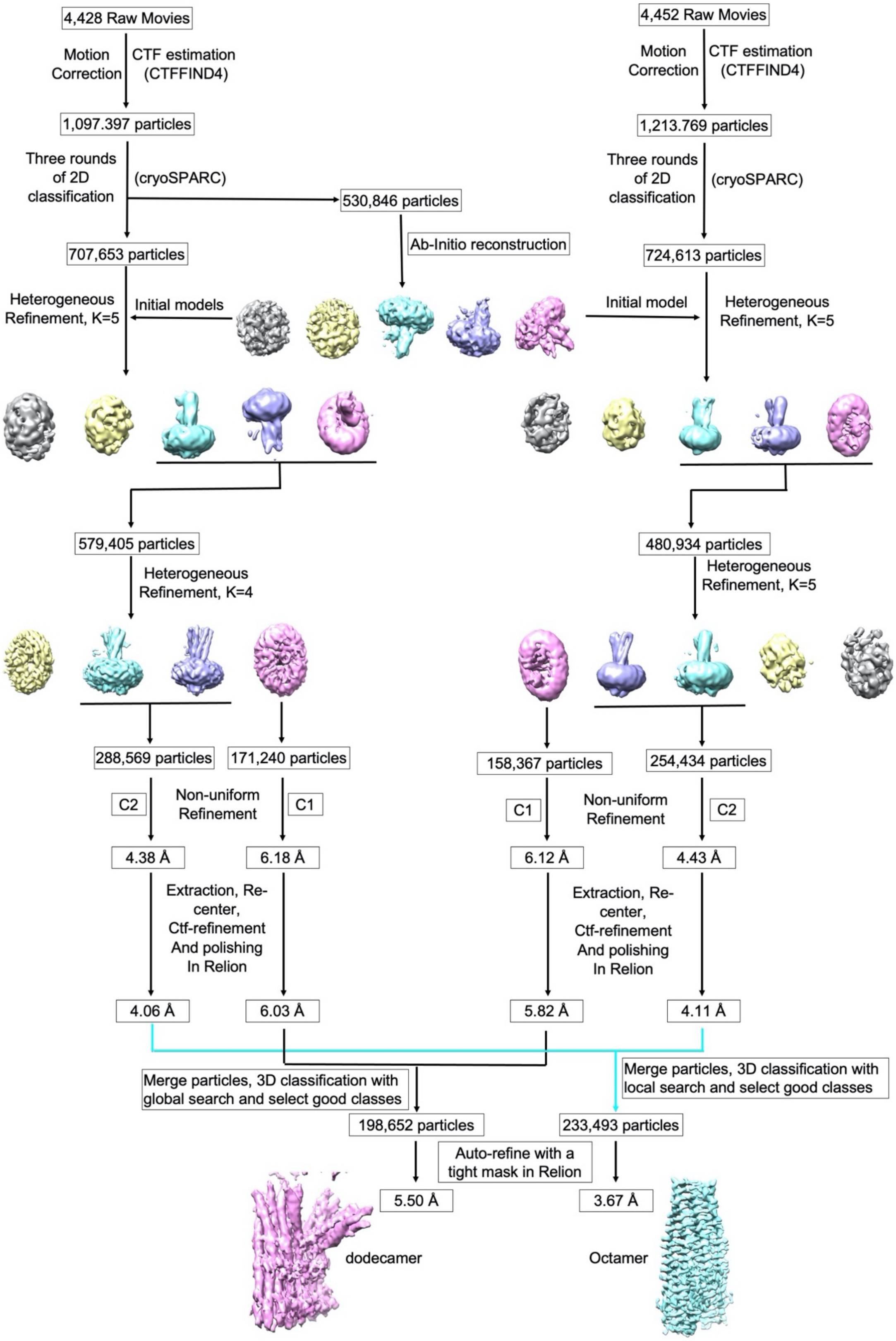

Fig. S2. Flow chart for cryo-EM data processing of TMED9. Data processing details can be found in Methods.

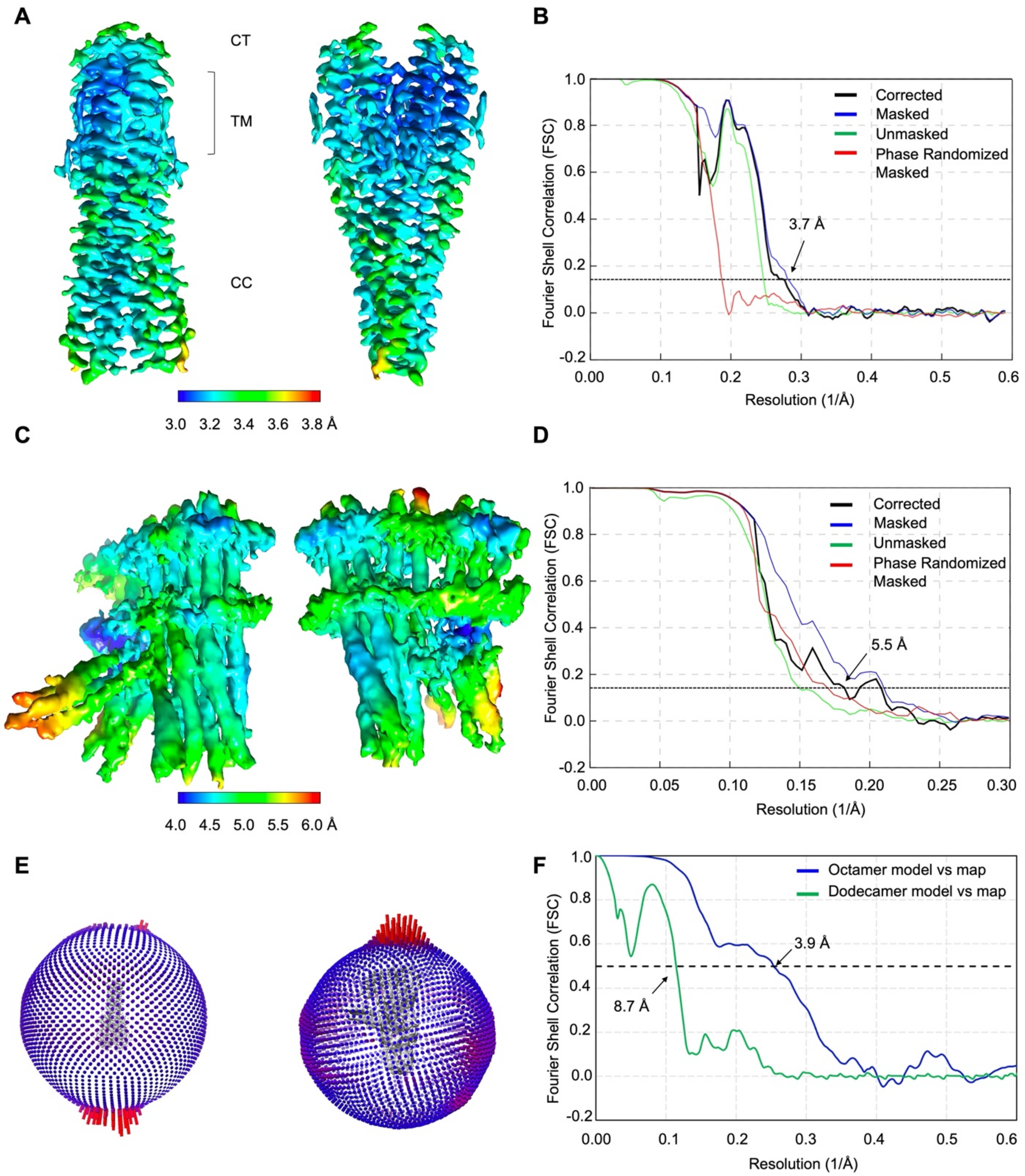

Fig. S3. Local resolution, FSC curves, angular distributions and validation of models. (**A-D**) Local resolution distributions of TMED9 octamer and dodecamer estimated using Phenix (A, C) and FSC curves for TMED9 octamer and dodecamer (B and D). The dashed lines represent FSC of 0.143. (**E**) Angular distribution plots for TMED9 octamer (left) and dodecamer (right) final maps. (**F**) FSC curves of the refined models versus final maps calculated for TMED9 Octamer (blue line) and TMED9 dodecamer (green line). The dashed lines represent FSC of 0.5.

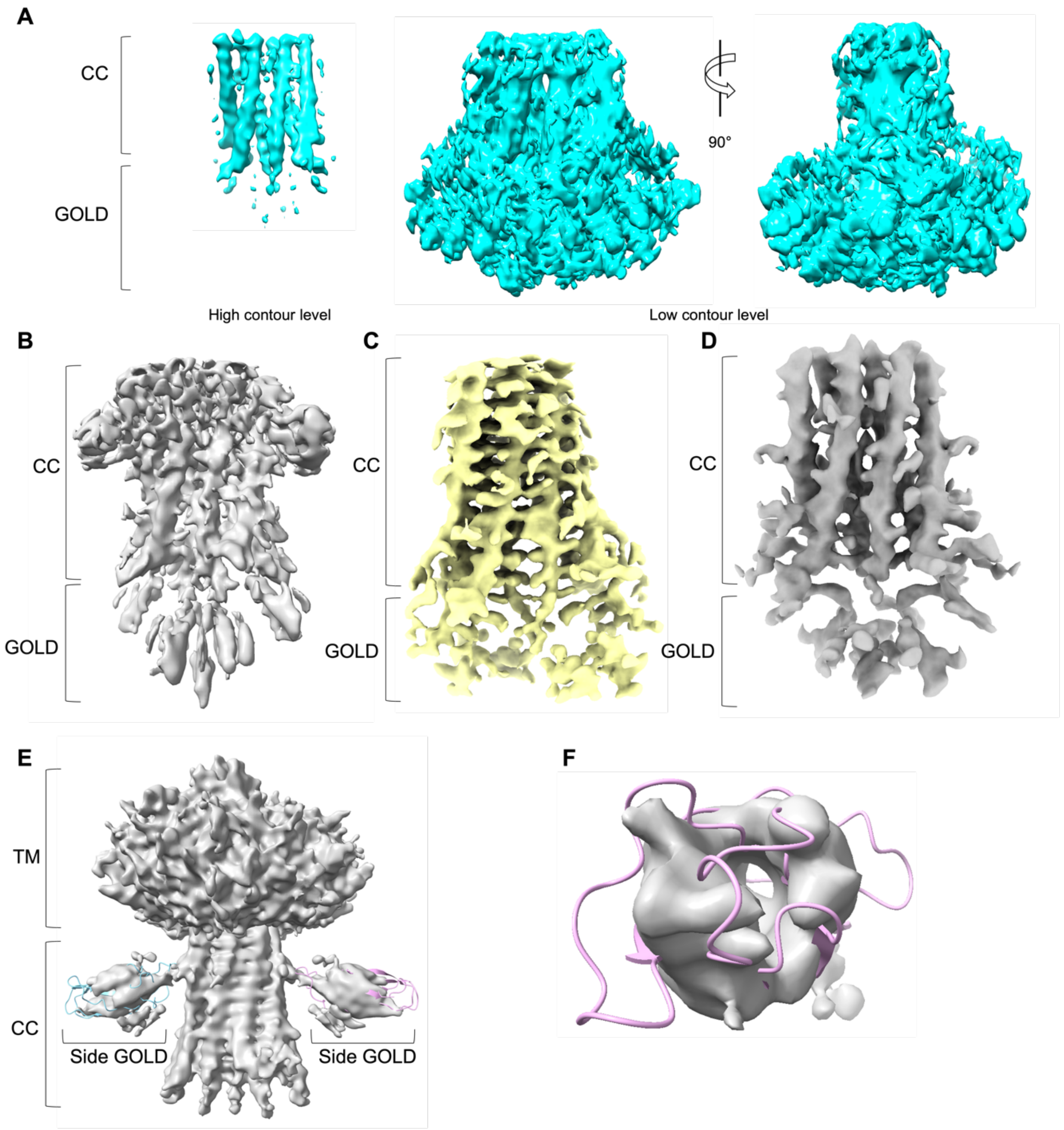

Fig. S4. TMED9 GOLD domain density. (A) Focused refinement of TMED9 CC+GOLD domain in CryoSparc resulting in a 4.8 Å resolution map showing strong density for the coiled coil domain and weak, diffuse density for the GOLD. **(B)** TMED9 CC+GOLD domain density map from CryoSparc after subtraction of the transmembrane domain and local refinement. **(C)** Density subtraction of the transmembrane domain followed by local refinement in Relion, resulting in a 4.3 Å resolution map. **(D)** The subtracted particles in Relion were applied to 3D classification without alignment and the class with the most particles was used for further local refinement, resulting in a map at 5 Å resolution. **(E)** Homogeneous Refinement of the octamer map from CryoSparc low pass filtered to 30 Å resolution generated a map at 4.4 Å resolution, which revealed two GOLD domains bound at the sides of the CC domain. The model of GOLD domain can be fitted into the map. **(F)** View of the right GOLD domain density superimposed with the GOLD domain model, showing the characteristic hole in the center of the domain.

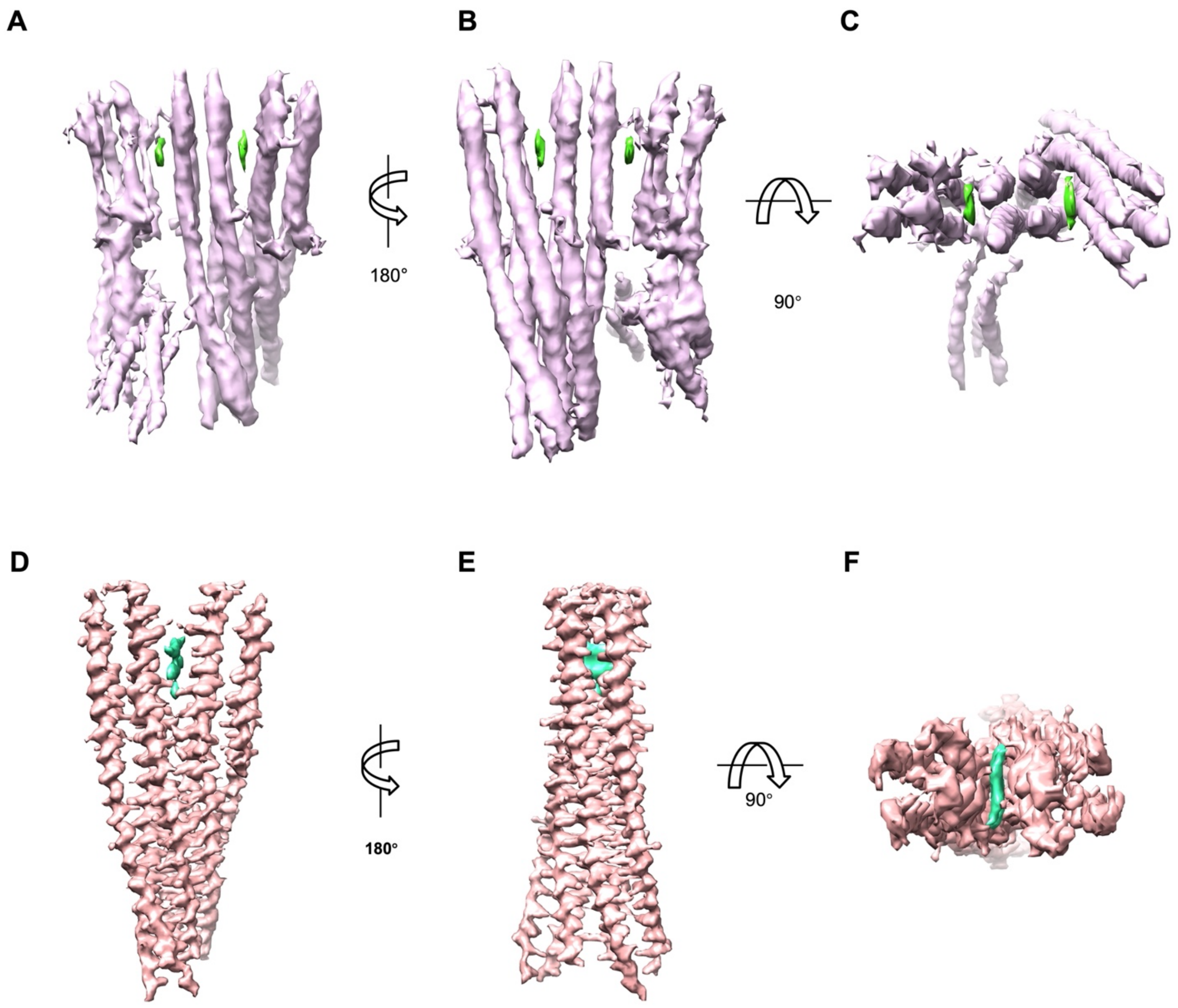

Fig. S5. Cryo-EM maps of TMED9 octamer and dodecamer, highlighting the lipid density. (**A-C**) Cryo-EM map of dodecameric TMED9 with the density for lipid shown in green. (**D-F**) Cryo-EM map of octameric TMED9 with the density for lipid shown in aquamarine.

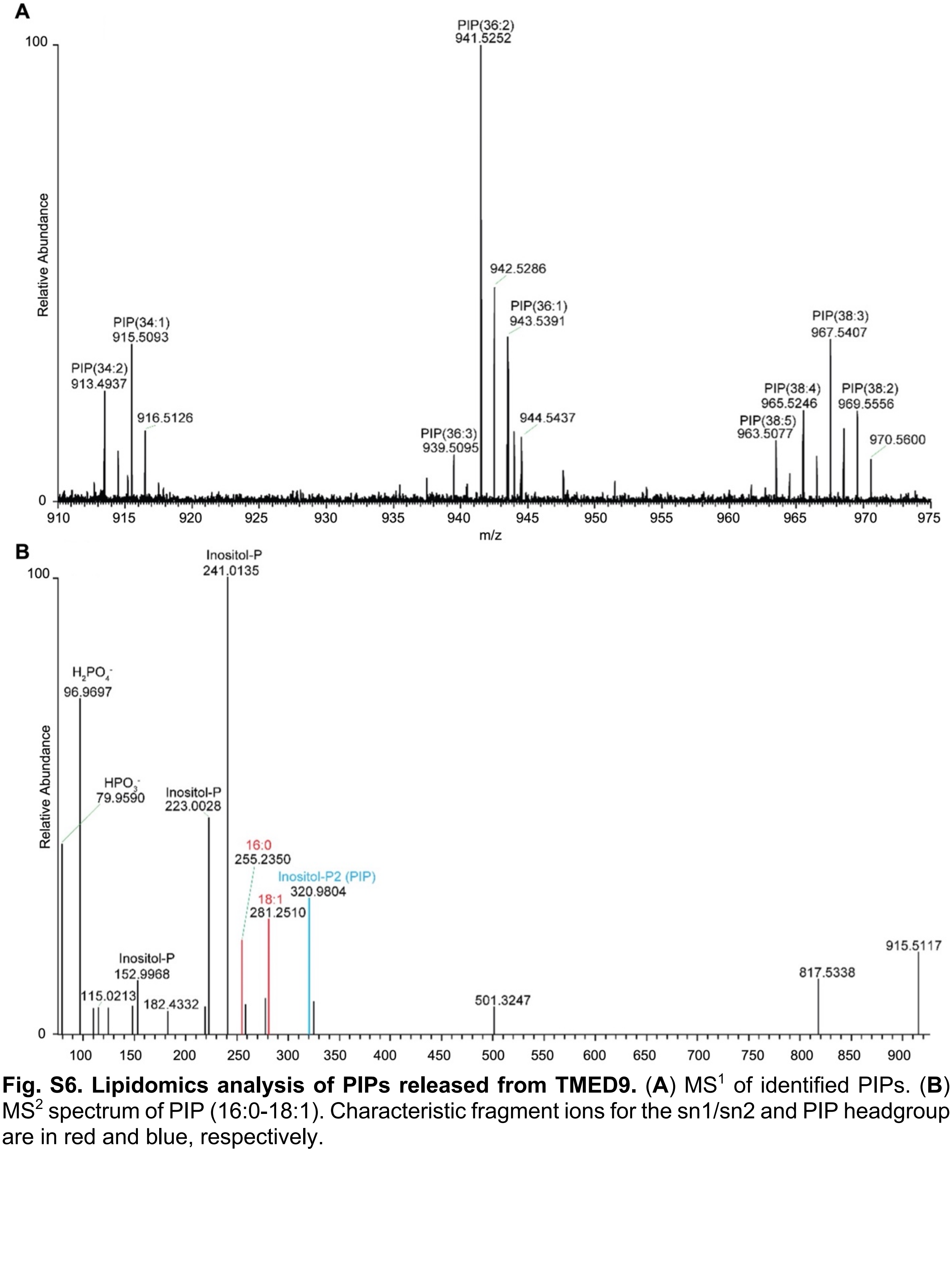

Fig. S6. Lipidomics analysis of PIPs released from TMED9. (**A**) MS^1 of identified PIPs. (**B**) MS^2 spectrum of PIP (16:0-18:1). Characteristic fragment ions for the sn1/sn2 and PIP headgroup are in red and blue, respectively.

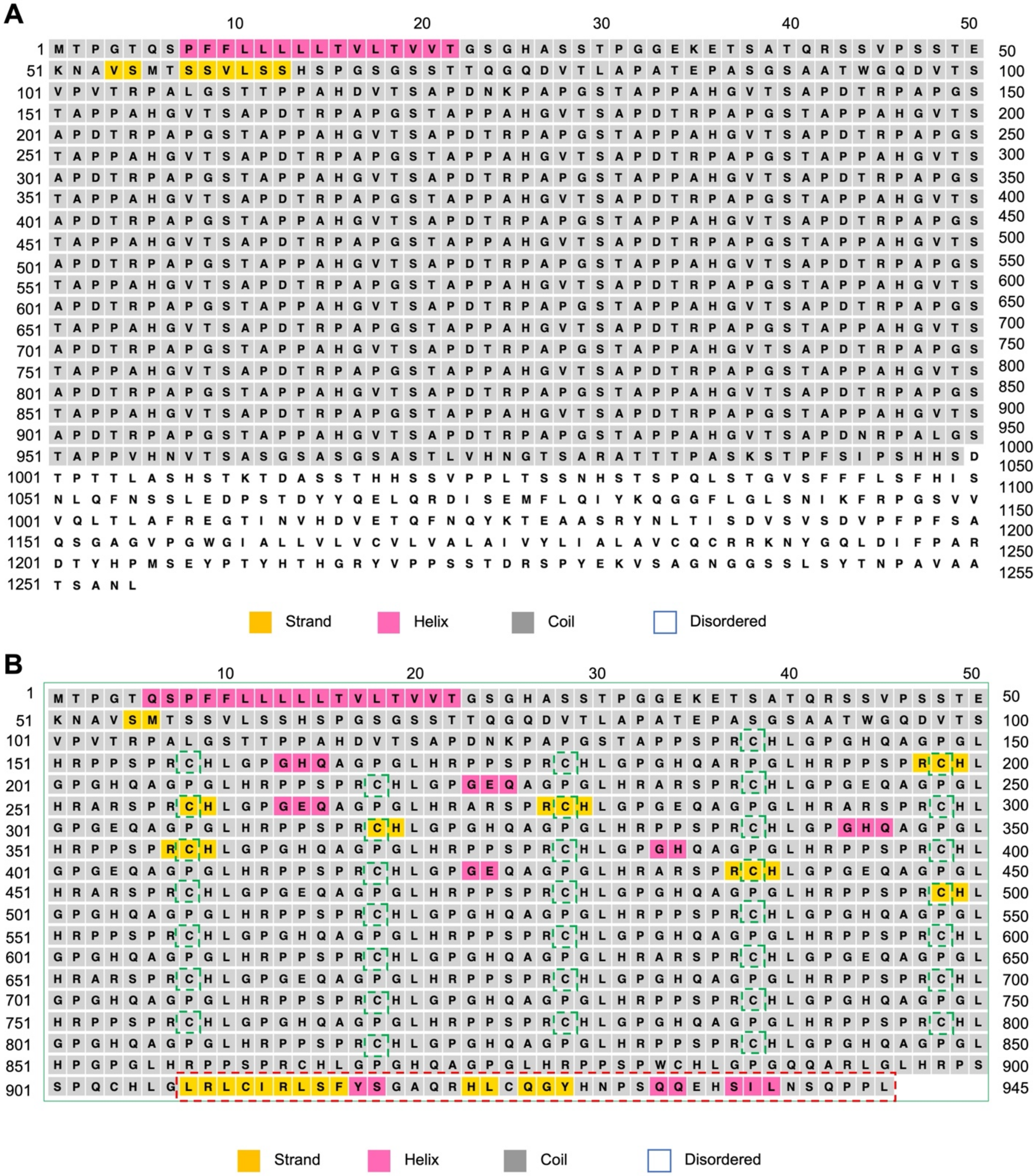

Fig. S7. Secondary structure prediction of MUC1 and MUC1-fs. (**A**) Secondary structure prediction of MUC1. (**B**) Secondary structure prediction of MUC1-fs. The dashed red box indicates the C-terminal ordered region (OR) of MUC1-fs, The dashed green boxes indicate the Cys residues in the long MUC1-fs repeat domain.

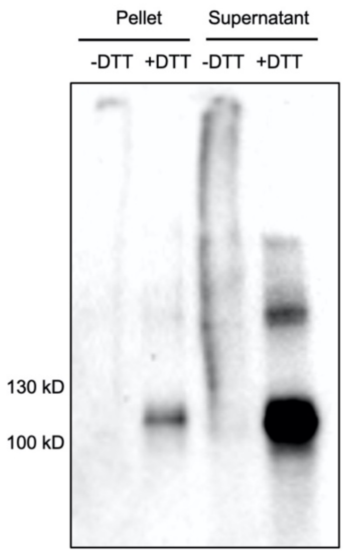

Fig. S8. Reducing and non-reducing SDS-PAGE of FLAG-tagged MUC1-fs, detected by anti-FLAG antibody.

A

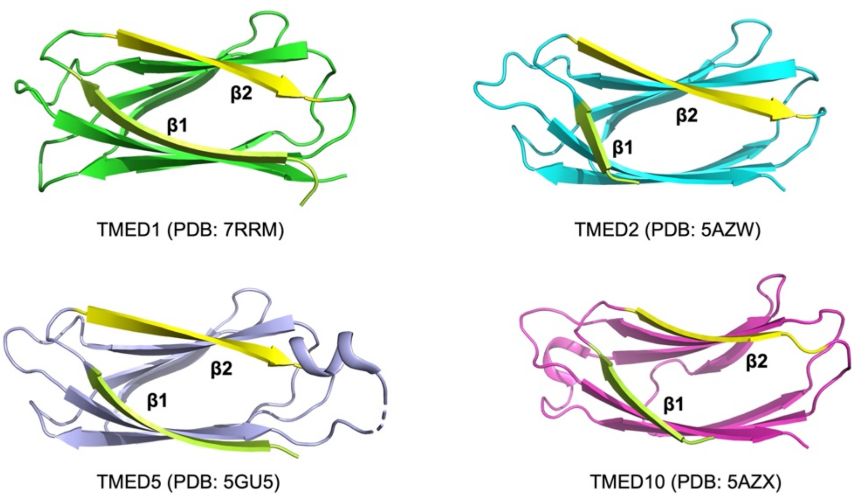

B

```
                     β1        β2
TMED9_GOLD  36  SALYFHIGETEKKCFIEEIPDETMVIGNYRTQLYDKQREEYQPATPGLGMFVEVKDPEDKVILAR 100
TMED10_GOLD 30  LAISFHLPINSRKCLREEIHK.....DLLVTGAYEISDQSGGAG..GLRSHLKITDSAGHILYSK 88
TMED7_GOLD  36   EITFELPDNAKQCFYEDIAQ.....GTKCTLEFQVITGGH......YDVDCRLEDPDGKVLYKE 88
TMED2_GOLD  19  SGYFVSIDAHAEECFFERVTS.....GTKMGLIFEVAEGGF......LDIDVEITGPDNKGIYKG 72
                               E52

TMED9_GOLD  101 QYGSEGRFTFTSHTPGEHQICLHSNSTKFSLFAGGMLRVHLDIQVGEHAND   151
TMED10_GOLD 89  EDATKGKFAFTTEDYDMFEVCFESK..GTGRIPDQLV..ILDMKHGVEAKN   134
TMED7_GOLD  89  MKKQYDSFTFTASKNGTYKFCFSN...EFSTFTHKTV..YFDFQVGEDPPLF  135
TMED2_GOLD  73  DRESSGKYTFAAHMDGTYKFCFSN...RMSTMTPKIV..MFTIDIGEAPK    118
```

Fig. S9. GOLD domains of TMEDs. (**A**) Crystal structures of TMEDs. The first (β1) and second (β2) β-stands are shown in lime and yellow, respectively. (**B**) Sequence alignment among the GOLD domains of TMED2, 7, 9 and 10, showing the conservation of E52 of TMED9 in β2.

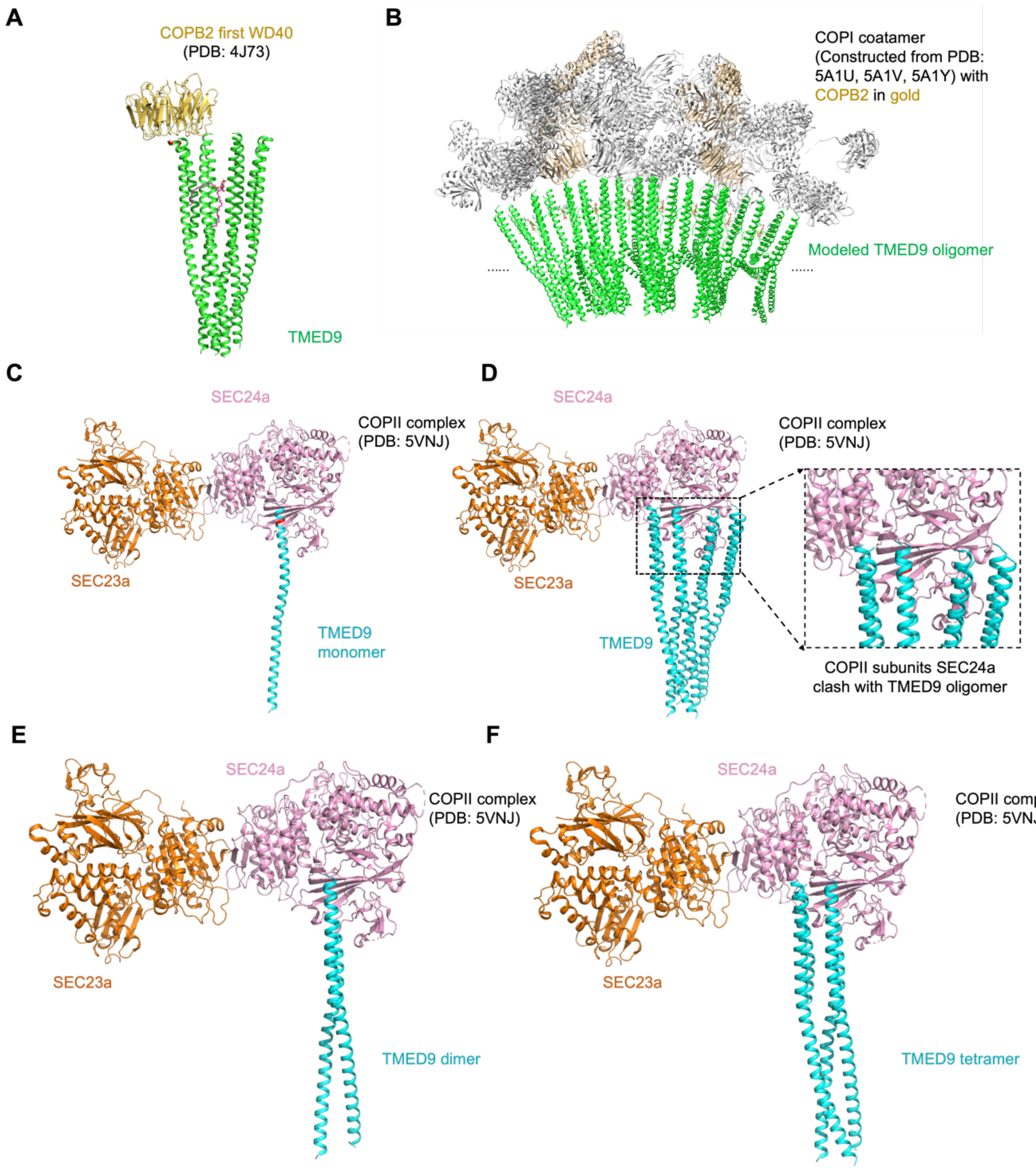

Fig. S10. Molecular modeling suggesting interaction of TMED9 with COPI but not COPII. (**A**) TMED9 octamer fitted to the first WD40 domain of COPB2 (a COPI component) in complex with the KK motif in a TMED9 tail peptide (PDB: 4J73). COPB2 and TMED9 tail peptide are shown in gold and red; TMED9 is shown in green with bound PIP (magenta). The black box indicates the fitted region around the KK motif of the TMED9 tail. (**B**) Modeled TMED9 higher-order oligomer in complex with a COPI coat (PDB: 5A1U, 5A1V, 5A1Y) showing two COPB2 subunits (gold) in contact with a TEMD9 oligomer. (**C-D**) TMED9 monomer (A) and octamer (D) fitted to the COPII SEC24a−SEC23a complex (pink and orange, respectively) in complex with the TMED9 FF motif (red) bound to ERGIC-53 (PDB: 5VNJ). TMED9 is shown in cyan. The black dashed boxes indicate the fitted region around the FF motif of the TMED9 tail. (**E-F**) TMED9 dimer (E) and tetramer (F) fitted to the same COPII SEC24a−SEC23a complex (pink and orange, respectively) in complex with the TMED9 FF motif (red) bound to ERGIC-53 (PDB: 5VNJ).

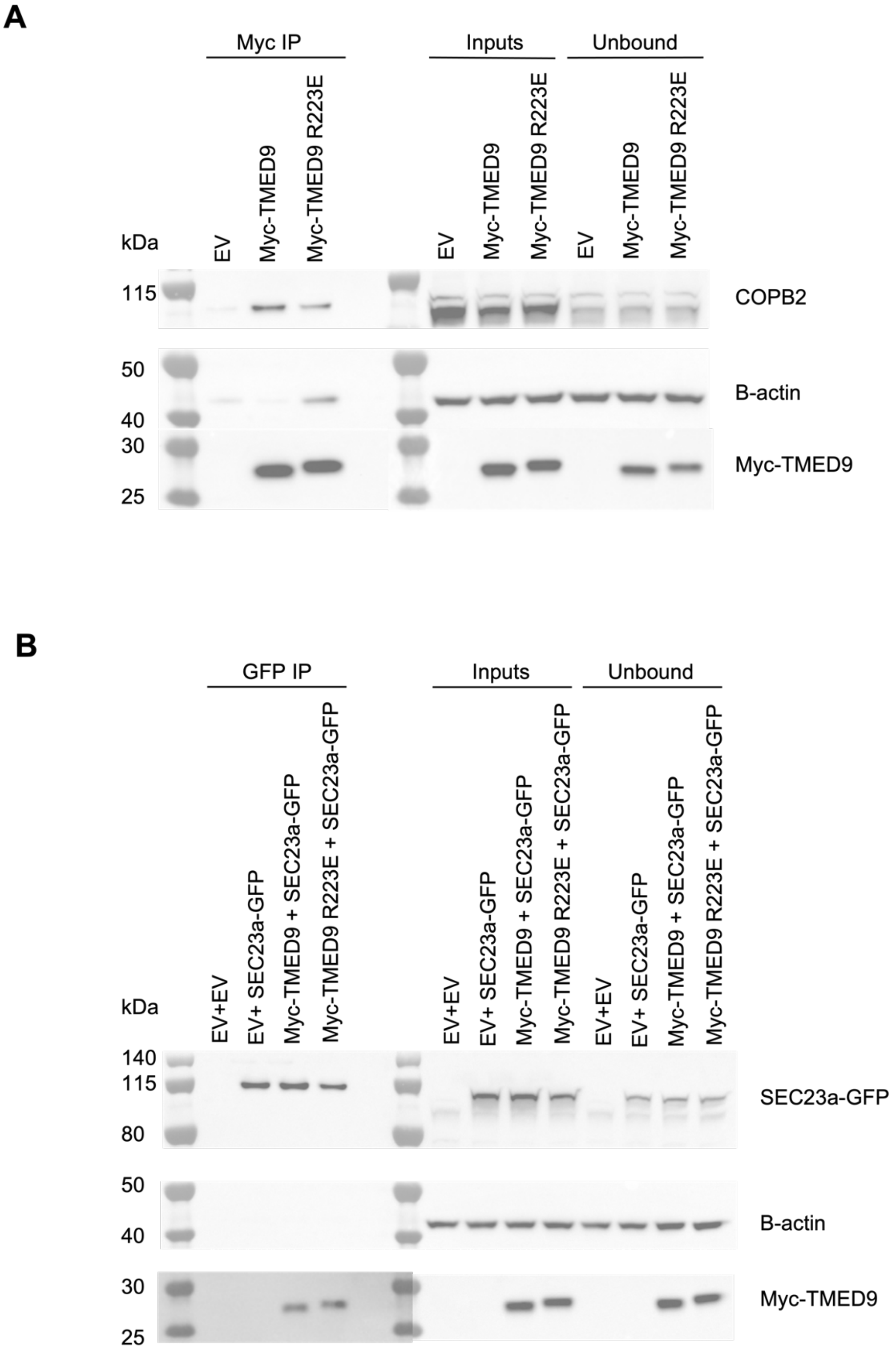

Fig. S11. co-IP of COP components with TMED9 WT and the R223E mutant. (**A)** Myc-tagged TMED9 and the R223E mutant coimmunoprecipitated COPB2. **(B)** GFP-tagged SEC23a coimmunoprecipitated WT and R223E mutant TMED9.

Table S1. Cryo-EM data collection, refinement, and validation statistics

	TMED9 (octamer)	TMED9 (dodecamer)
**Data collection and processing**		
Nominal Magnification	105,000	105,000
Voltage (keV)	300	300
Electron exposure ($e^-/Å^2$)	58.8	58.8
Defocus range (μm)	-1.5 − -2.2	-1.5 − -2.2
Pixel size (Å)	0.825	0.825
Symmetry imposed	C2	C1
Initial particle images (no.)	2,311,166	2,311,166
Final particle images (no.)	233,493	198,652
Map resolution (Å)	3.7	5.5
FSC threshold	0.143	0.143
Map resolution range (Å)	3.0 – 3.8	4.0 – 6.0
**Refinement**		
Initial model used	AlphaFold	AlphaFold
Model resolution (Å)	3.7	5.5
FSC threshold	0.143	0.143
Map sharpening *B* factor ($Å^2$)	-171	-263
Model composition		
Non-hydrogen atoms	4,781	8,053
Protein residues	544	908
Ligands	1	2
B-factors($Å^2$)		
Protein	125.37	102.42
Ligand	20	20
R.m.s. deviations		
Bond lengths (Å)	0.021	0.006
Bond angles (º)	1.928	1.163
Validation		
MolProbity score	2.47	3.30
Clashscore	28.33	48.86
Poor rotamers (%)	5.04	18.59
Ramachandran Plot		
Favored (%)	99.05	97.06
Allowed (%)	0.76	1.92
Disallowed (%)	0.19	1.02

Table S2. Summary of PIP lipids identified by lipidomics. The first and second numbers in sn1 and sn2 denote acyl carbon length and the number of double bonds, respectively.

Lipid ID	sn1[a]	sn2[a]	Observed (Da)	Theoretical (Da)	Mass Error
PIP(34:2)	16-1	18-1	914.5012	914.4929	8.3 ppm
PIP(34:1)	16-0	18-1	916.5171	916.5085	8.6 ppm
PIP(36:3)	18-1	18-2	940.5183	940.5085	9.8 ppm
PIP(36:2)	18-1	18-1	942.5330	942.5242	8.8 ppm
PIP(38:5)	-	-	964.5158	964.5085	7.3 ppm
PIP(38:4)	20-3	18-1	966.5328	966.5242	8.6 ppm
PIP(38:3)	20-2	18-1	968.5477	968.5398	7.9 ppm
PIP(38:2)	20-2,20-1	18-0,18-1	970.5643	970.5555	8.8 ppm

[a] if annotated, at least one signal for the sn1 or sn2 alkyl chain was identified in the MS^2 scan.
